# RTL1/PEG11 imprinted in human and mouse brain mediates anxiety-like and social behaviors and regulates neuronal excitability in the locus coeruleus

**DOI:** 10.1093/hmg/ddac110

**Published:** 2022-05-14

**Authors:** Ming-Yi Chou, Meng-Chuen Hu, Pin-Yu Chen, Chi-Lin Hsu, Ting-Yu Lin, Mao-Jia Tan, Chih-Yu Lee, Meng-Fai Kuo, Pei-Hsin Huang, Vin-Cent Wu, Shih-Hung Yang, Pi-Chuan Fan, Hsin-Yi Huang, Schahram Akbarian, Tsui-Han Loo, Colin L Stewart, Hsiang-Po Huang, Susan Shur-Fen Gau, Hsien-Sung Huang

**Affiliations:** Graduate Institute of Brain and Mind Sciences, College of Medicine, National Taiwan University, Taipei 10051, Taiwan; Graduate Institute of Brain and Mind Sciences, College of Medicine, National Taiwan University, Taipei 10051, Taiwan; Graduate Institute of Brain and Mind Sciences, College of Medicine, National Taiwan University, Taipei 10051, Taiwan; Graduate Institute of Brain and Mind Sciences, College of Medicine, National Taiwan University, Taipei 10051, Taiwan; Graduate Institute of Brain and Mind Sciences, College of Medicine, National Taiwan University, Taipei 10051, Taiwan; Graduate Institute of Brain and Mind Sciences, College of Medicine, National Taiwan University, Taipei 10051, Taiwan; Graduate Institute of Brain and Mind Sciences, College of Medicine, National Taiwan University, Taipei 10051, Taiwan; Graduate Institute of Medical Genomics and Proteomics, College of Medicine, National Taiwan University, Taipei 10051, Taiwan; Division of Neurosurgery, Department of Surgery, National Taiwan University Hospital and National Taiwan University College of Medicine, Taipei 10051, Taiwan; Department of Pathology, National Taiwan University Hospital and College of Medicine, National Taiwan University, Taipei 10051, Taiwan; Department of Internal Medicine, National Taiwan University Hospital and College of Medicine, National Taiwan University, Taipei 10051, Taiwan; Division of Neurosurgery, Department of Surgery, National Taiwan University Hospital and National Taiwan University College of Medicine, Taipei 10051, Taiwan; Department of Pediatrics, National Taiwan University Hospital and College of Medicine, National Taiwan University, Taipei 10051, Taiwan; Department of Pathology, National Taiwan University Hospital and College of Medicine, National Taiwan University, Taipei 10051, Taiwan; Department of Psychiatry, Icahn School of Medicine at Mount Sinai, NY 10029, USA; A^*^STAR Skin Research Labs, Agency for Science, Technology and Research, Singapore 138632, Singapore; A^*^STAR Skin Research Labs, Agency for Science, Technology and Research, Singapore 138632, Singapore; Graduate Institute of Medical Genomics and Proteomics, College of Medicine, National Taiwan University, Taipei 10051, Taiwan; Graduate Institute of Brain and Mind Sciences, College of Medicine, National Taiwan University, Taipei 10051, Taiwan; Department of Psychiatry, National Taiwan University Hospital and College of Medicine, National Taiwan University, Taipei 10051, Taiwan; Graduate Institute of Brain and Mind Sciences, College of Medicine, National Taiwan University, Taipei 10051, Taiwan

## Abstract

*RTL1/PEG11*, which has been associated with anxiety disorders, is a retrotransposon-derived imprinted gene in the placenta. However, imprinting patterns and functions of RTL1 in the brain have not been well-investigated. We found *Rtl1* was paternally, but not maternally, expressed in brain stem, thalamus, and hypothalamus of mice, and imprinting status of *RTL1* was maintained in human brain. Paternal *Rtl1* knockout (*Rtl1^m+/p-^*) mice had higher neonatal death rates due to impaired suckling, and low body weights beginning on embryonic day 16.5. High paternal expression of *Rtl1* was detected in the locus coeruleus (LC) and *Rtl1^m+/p-^* mice showed an increased delay in time of onset for action potentials and inward currents with decreased neuronal excitability of LC neurons. Importantly, *Rtl1^m+/p-^* mice exhibited behaviors associated with anxiety, depression, fear-related learning and memory, social dominance, and low locomotor activity. Taken together, our findings demonstrate RTL1 is imprinted in brain, mediates emotional and social behaviors, and regulates excitability in LC neurons.

## Introduction

Retrotransposon Gag like 1 (*RTL1*), which is also known as Paternally expressed gene 11 (*PEG11*), is a paternally-, but not maternally expressed imprinted gene in placentas and embryos (1,2) and is essential for placental permeability in the mid-to-late fetal stages (3,4). Importantly, using a broad anxiety phenotype rather than a DSM-IV anxiety disorder diagnosis, a linkage study suggested a region on chromosome 14 could contain genes associated with anxiety in humans, and emotionality in mice, which contains *RTL1* (5). However, whether *Rtl1* is imprinted in the brain and whether RTL1 plays a role in anxiety-like or emotional behaviors has not been well-investigated. Thus, the emerging questions are what imprinting patterns of *RTL1* are in mouse and human brain, whether there is a causal relationship between RTL1 dysfunction and anxiety, and how RTL1 contributes to anxiety. Answers to the above questions could lead to a better understanding of the imprinting status and functional roles of RTL1 in the brain as well as in anxiety disorders.

Here, we used *Rtl1* knockout mice as a model to identify imprinting patterns of *Rtl1* in mouse brain, and behavioral and neuronal roles of RTL1. We also used human postmortem and surgical resection brains to identity imprinting patterns of *RTL1* in human brain. Our results provide not only compelling evidence that loss of *Rtl1* is responsible for mediating anxiety-like and social behaviors but also how the brain governs anxiety-like and social behaviors in addition to imprinting maps of *Rtl1* in brain.

## Results

### Comprehensive profiling of *Rtl1* expression in mice

**Figure 1:**
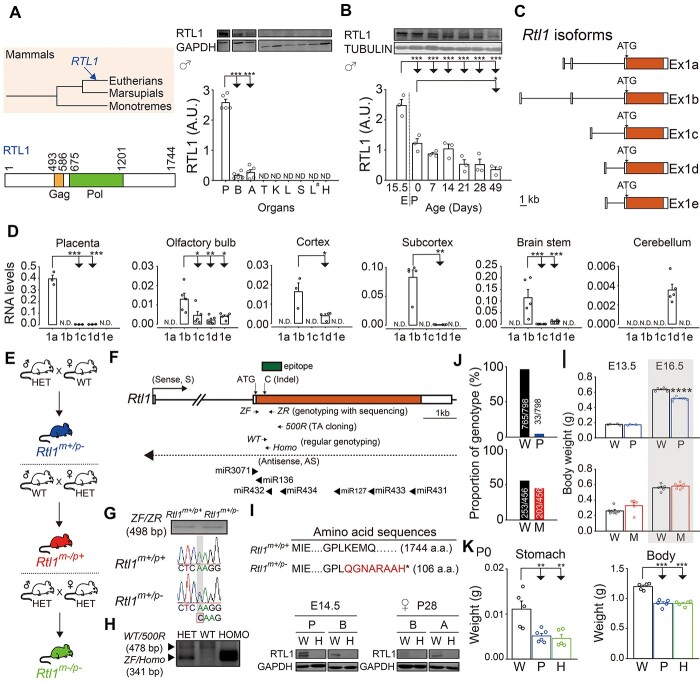
Expression profiling of *Rtl1* and validation of *Rtl1* knockout mice. (**A**) Schematic of the presence of *RTL1* in the evolutionary tree of mammals (left, top). Schematic of the functional domain of mouse RTL1 protein. GAG and POL are two major proteins encoded within the retroviral genome (left, bottom). Levels of RTL1 protein were measured with western blot analysis from different organs of male postnatal day 28 (P28) mice. RTL1 protein levels were normalized to GAPDH protein. One-way ANOVA with Holm-Sidak *post hoc* comparison. *n* = 5 per group. P = placenta, B = brain, A = adrenal gland, T = testis, K = kidney, L = lung, S = spleen, L^#^ = liver, H = heart. ND = non-detectable. (**B**) Levels of RTL1 protein were measured with western blot analysis from embryonic stage (embryonic day 15.5) to adult stage (postnatal day 49) of male mice. RTL1 protein levels were normalized to alpha-TUBULIN protein. One-way ANOVA with Holm-Sidak *post hoc* comparison. *n* = 3 brain hemispheres for each time points. (**C**) Schematic of *Rtl1* isoforms based on a previous finding (2). Location of ATG start codon is indicated. Scale bar = 1 kb. (**D**) Five *Rtl1* transcript isoforms were analyzed with qPCR from placenta (E15.5 pups), and five brain regions of P28 male mice (olfactory bulb, cortex, subcortex, brain stem, and cerebellum). Student’s *t*-test, two-tailed, Mann–Whitney rank sum test, or one-way ANOVA with Holm-Sidak *post hoc* comparison. *n* = 3–5 for each isoform. (**E**) *Rtl1^m+/p-^* mice were generated by mating male *Rtl1* heterozygous knockout mice (HET) with female wild-type mice (WT). *Rtl1^m−/p+^* mice were generated by mating male WT mice with female HET mice. *Rtl1^m−/p-^* mice were generated by mating male HET mice with female HET mice. (**F**) Schematic of *Rtl1* genomic locus. Locations of ATG start codon, Indel site, primers, miRNAs, and epitope for RTL1 antibody are indicated. (**G**) Sanger sequencing of tail genomic DNA from WT and *Rtl1^m+/p-^* mice using *ZF* and *ZR* primers (top). Nucleotide sequences of CRISPR-edited *Rtl1* loci for WT and *Rtl1^m+/p-^* mice (bottom). (**H**) Regular genotyping by PCR analysis of tail genomic DNA from HET, WT, and HOMO mice using *WT* and *500R* primers for detecting wild-type allele, and *ZF* and *Homo* primers for detecting mutant allele. (**I**) Amino acid sequences of CRISPR-edited *Rtl1* loci for WT and *Rtl1^m+/p-^* mice (top). Levels of RTL1 protein were measured with western blot analysis from E14.5 placenta (P) and brain (B), and P28 brain (B) and adrenal gland (A) of *Rtl1* homozygous knockout mice (H) and their corresponding WT controls (W) (bottom). GAPDH protein was used as a loading control. (**J**) Proportion of genotypes of *Rtl1^m+/p-^* (P), *Rtl1^m−/p+^* (M) mice, and their corresponding wild-type (W) mice littermates were calculated at P28. (**K**) Stomach and body weights were measured from *Rtl1^m−/p+^* (P), *Rtl1^m−/p-^* (H), and wild-type (WT) mice at postnatal day 0 (P0). One-way ANOVA with Holm-Sidak *post hoc* comparison. *n* = 5–6 per group. (**L**) Body weights from *Rtl1^m+/p-^* (P) (top), *Rtl1^m−/p+^* (M) (bottom) mice, and their corresponding wild-type controls (W) at embryonic day 13.5 (E13.5) and E16.5. Student’s *t*-test, two-tailed. *n* = 3–6 per group. ^*^P < 0.05, ^*^^*^P < 0.01, ^*^^*^^*^P < 0.001, ^*^^*^^*^^*^P < 0.0001. All data are the mean ± SEM.

*Rtl1* is a eutherian-specific and retrotransposon-derived gene (6) (Fig. 1A, left). Previous studies focused on the expression patterns of *Rtl1* sense and antisense (7–9). However, expression patterns of RTL1 protein have not been well characterized. To address this question, we first profiled RTL1 protein expression in different organs of male mice at postnatal day 28 (P28) with western blot analysis. RTL1 protein was predominantly expressed in brain and adrenal gland (Fig. 1A, right). Expression of RTL1 protein in placenta was used as a positive control due to its specificity to eutherian mammals (Fig. 1A, right). To further investigate temporal patterns of RTL1 protein expression with a focus on brain, we performed western blot analysis from brains of male mice from embryonic day 15.5 (E15.5) to postnatal day 49 (P49). Higher expression of RTL1 protein occurred in the brain of embryonic and neonatal mice (Fig. 1B). Expression patterns of RTL1 protein in organs and brains from female mice (Supplementary Material, Fig. S1) were consistent with male mice. *Rtl1* in mice has five transcript isoforms (Fig. 1C) (2) and their expression patterns have not been examined in brain. To address this issue, we determined expression levels of the five isoforms of the *Rtl1* transcript in mouse brain using quantitative PCR, with a focus on the olfactory bulb, cortex, subcortex, brain stem and cerebellum based on a previous finding showing *Rtl1* was expressed in these five brain regions (9) using mouse placenta as a control tissue. Here, we found *Rtl1 Ex1b* was predominantly expressed in the olfactory bulb, cortex, subcortex and brain stem of male mice at P28 (Fig. 1D). *Rtl1 Ex1d* was predominantly expressed in the cerebellum of male mice at P28 (Fig. 1D). *Rtl1 Ex1a* was predominantly expressed in the placenta of mice (Fig. 1D), which is consistent with a previous finding (2). These data showed specific expression of *Rtl1* in organs and brains of mice, which varied with stages of development from embryo to adult.

### Validation of *Rtl1* knockout mice

To fully confirm the expression and imprinting patterns of *Rtl1* in mouse brain, mice were mated to generate paternal (*Rtl1^m+/p-^*), maternal (*Rtl1^m−/p+^*) and homozygous (*Rtl1^m−/p-^*) *Rtl1* knockout mice (Fig. 1E). There are seven microRNAs within the coding exon of *Rtl1* in mice (Fig. 1F). To specifically delete *Rtl1* in mice without disturbing nearby microRNAs, we created an indel mutation in the coding exon of *Rtl1* with a CRISPR approach (Fig. 1F). Our *Rtl1* knockout mice were verified by Sanger sequencing (Fig. 1G), genotyping (Fig. 1H), western blot analysis (Fig. 1I) and TA cloning of F1 generation (Supplementary Material, Fig. S2).

Comparing genotypes of littermates at P28 from mating pairs generating *Rtl1^m+/p-^* mice, 4% of littermates were *Rtl1^m+/p-^* (P) mice (33/798; male = 17, female = 16) and 96% of littermates were wild-type (W) mice (765/798; male = 401, female = 364) (Fig. 1J, top). In contrast, *Rtl1^m−/p+^* (M) mice at P28 represented 45% of their littermates from mating pairs generating *Rtl1^m−/p+^* mice (203/456; male = 97, female = 106), and wild-type (W) mice at P28 represented 55% of their littermates (253/456; male = 119, female = 134) (Fig. 1J, bottom). The percentage of *Rtl1^m+/p-^* mice among littermates was lower compared with their wild-type counterpart (Fig. 1J, top).

Decreased stomach weight in *Rtl1^m+/p-^* mice (Fig. 1K, left) is an indicator of inadequate milk consumption, which suggests the mice had difficulties with suckling and could explain the high rate of death in neonatal *Rtl1^m+/p-^* mice (Fig. 1J, top). Decreased body weight in *Rtl1^m+/p-^* but not *Rtl1^m−/p+^* mice was detectable at E16.5 (Fig. 1L), and this pattern continued until at least P49 (P0, Fig. 1K, right; P28, Supplementary Material, Fig. S3A; from P7 to P49, Supplementary Material, Fig. S4). Decreased placenta weight in *Rtl1^m+/p-^* but not *Rtl1^m−/p+^* mice was also detectable at E16.5 (Supplementary Material, Fig. S5). More importantly, *Rtl1^m−/p-^* mice showed decreased brain weight (Supplementary Material, Fig. S3B) and increased ratio of brain to body weight (Supplementary Material, Fig. S3C). Our results indicate the successful generation of *Rtl1* knockout mice with significant phenotypes in *Rtl1^m+/p-^* mice.

### *Rtl1* is paternally expressed in mouse brain stem, thalamus and hypothalamus

**Figure 2:**
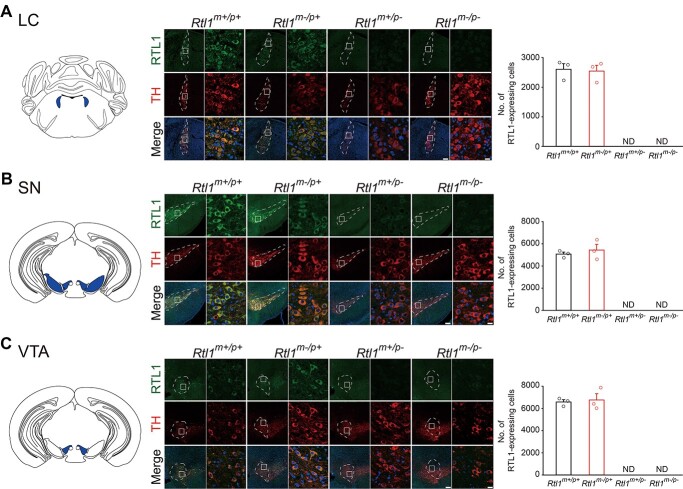
*Rtl1* is paternally, but not maternally, expressed in tyrosine hydroxylase (TH)-expressing brain regions of mice. Schematic diagram of TH-expressing brain regions containing paternally-expressed RTL1: (**A**, left) LC, (**B**, left) substantia nigra (SN) and (**C**, left) ventral tegmental area (VTA). (A–C, middle) Representative sections from mouse brains immunostained with RTL1 antibody (green), TH antibody (red) and counter-stained with DAPI (blue) in *Rtl1^m+/p+^*, *Rtl1^m−/p+^*, *Rtl1^m+/p-^* and *Rtl1^m−/p-^* mice. Scale bar = 150 μm. Squares indicate areas enlarged in left panel for each mouse genotype. Scale bar = 15 μm. (A–C, right). Bar graphs indicate the number of RTL1-expressing cells counted in brain regions of *Rtl1^m+/p+^*, *Rtl1^m−/p+^*, *Rtl1^m+/p-^* and *Rtl1^m−/p-^* mice. *n* = 3 per group. ND = non-detectable. All data are the mean ± SEM.

To comprehensively profile the expression patterns and imprinting status of *Rtl1* in mouse brain, we performed immunofluorescence staining with an antibody against RTL1 in P49 brain sections from *Rtl1^m−/p+^*, *Rtl1^m+/p-^* and *Rtl1^m−/p-^* mice and their corresponding WT (*Rtl1^m+/p+^*) controls. We found *Rtl1* was abundantly expressed paternally, but not maternally, in the locus coeruleus (LC) (Fig. 2A), substantia nigra (SN) (Fig. 2B) and ventral tegmental area (VTA) (Fig. 2C), which are all tyrosine hydroxylase (TH)-expressing regions of the brain stem. In addition, *Rtl1* was paternally, but not maternally expressed in other TH-expressing regions of the brain stem: the dorsal raphe nucleus (DRC) (Supplementary Material, Fig. S6A), and retrorubral field (RRF) (Supplementary Material, Fig. S6B). TH-positive neurons of RRF project to the accumbens nucleus core (AcbC) in which *Rtl1* was paternally, but not maternally, expressed (Supplementary Material, Fig. S6C). *Rtl1* was also expressed in a TH-expressing bundle, the medial forebrain bundle (MFB), which connects the olfactory bulb to the brain stem (Supplementary Material, Fig. S6D). We also examined whether *Rtl1* is expressed in periglomerular neurons of the olfactory bulb, which has a particularly high level of TH expression, and determined *Rtl1* is not expressed in this cell type (Supplementary Material, Fig. S7). Paternal but not maternal expression of *Rtl1* was also detected in several non-TH-expressing regions of the brain stem (Supplementary Material, Fig. S8B–K), the paraventricular nucleus of the thalamus (PVT) (Supplementary Material, Fig. S8L) and zona incerta, caudal (ZIC) in the thalamus (Supplementary Material, Fig. S8M), and periventricular nucleus (PVN) in the hypothalamus (Supplementary Material, Fig. S8N).

**Figure 3:**
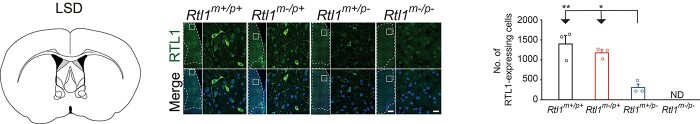
The imprinting status of *Rtl1* is relaxed in regions of lateral septal nucleus, dorsal part (LSD). (Left) Schematic diagram of a brain region of mouse demonstrating biallelic *Rtl1* expression in the LSD. (Middle) Representative sections from mouse LSD immunostained with RTL1 antibody (green), and counter-stained with DAPI (blue) in *Rtl1^m+/p+^*, *Rtl1^m−/p+^*, *Rtl1^m+/p-^*, and *Rtl1^m−/p-^* mice. Scale bar = 150 μm. Squares indicate areas enlarged in left panel for each mouse genotype. Scale bar = 15 μm. (Right) Bar graphs indicate the number of RTL1-expressing cells counted in the LSD of *Rtl1^m+/p+^*, *Rtl1^m−/p+^*, *Rtl1^m+/p-^* and *Rtl1^m−/p-^* mice. One-way ANOVA with Tukey *post hoc* comparison, ^*^P < 0.05, ^*^^*^P < 0.01. *n* = 3 per group. ND = non-detectable. All data are the mean ± SEM.

**Figure 4:**
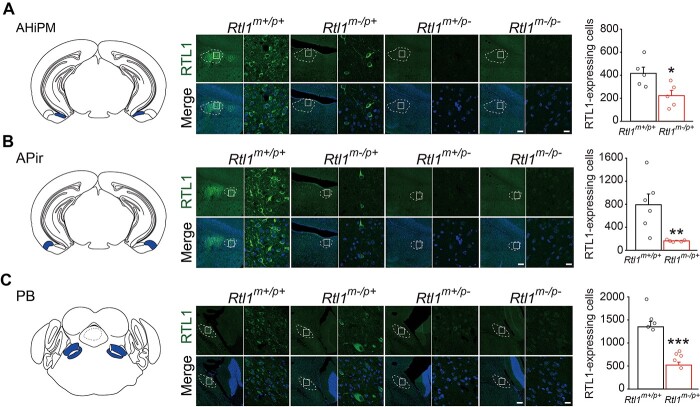
Maternal *Rtl1* knockout mice display fewer RTL1-expressing cells in three brain regions compared with wild-type mice. (**A–C,** left) Schematic diagrams of brain regions examined (amygdalohippocampal area, posteromedial part (AHiPM), amygdalopiriform transition (APir) and parabrachial nuclei (PB), respectively). (A–C, middle) Representative sections of mouse brains immunostained with RTL1 demonstrating fewer RTL1-expressing cells in *Rtl1^m−/p+^* mice: (A) AHiPM, (B) APir and (C) PB. Scale bar = 150 μm. Squares indicate areas enlarged in left panel for each mouse genotype. Scale bar = 15 μm. (A–C, right) Bar graphs indicate the number of RTL1-expressing cells counted in the three brain regions of *Rtl1^m+/p+^*, and *Rtl1^m−/p+^* mice. Student’s *t*-test, two-tailed, or Mann–Whitney rank sum test, ^*^P < 0.05, ^*^^*^P < 0.01, ^*^^*^^*^P < 0.001. *n* = 5–6 per group. All data are the mean ± SEM.

Furthermore, *Rtl1* relaxed its imprinting status in regions of the lateral septal nucleus, dorsal part (LSD) (Fig. 3). Although *Rtl1* was paternally, but not maternally, expressed in the amygdalohippocampal area, posteromedial part (AHiPM) (Fig. 4A), amygdalopiriform transition (APir) (Fig. 4B) and parabrachial nuclei (PB) (Fig. 4C), all three brain regions showed fewer RTL1-expressing cells in *Rtl1^m−/p+^* mice compared with WT mice (Fig. 4A–C, right). Our results suggest *Rtl1* is paternally, but not maternally, expressed in mouse brain with a relaxation of imprinting in the LSD. In addition, the major cell types for RTL1-positive and TH-negative cells in mouse brain are neurons (Supplementary Material, Figs S9–S11).

### *RTL1* is imprinted in human brain and adrenal gland

**Figure 5:**
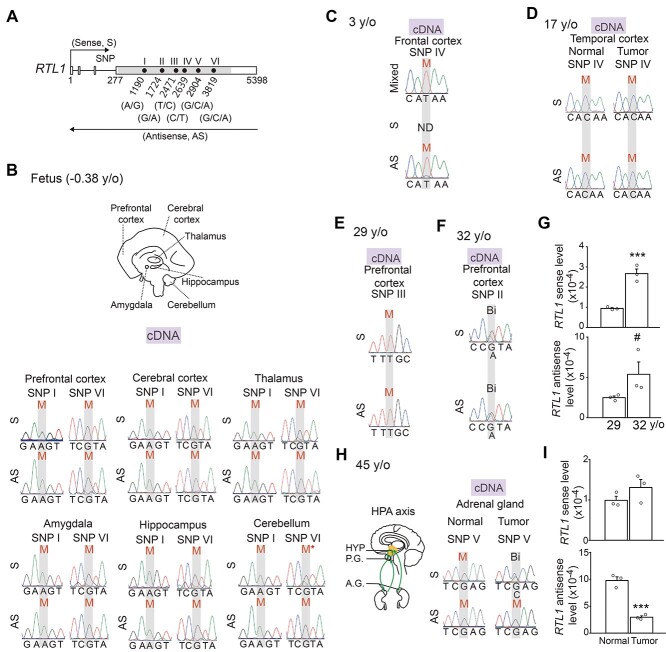
Imprinting status of *RTL1* is maternally, but not paternally, expressed in human brain and adrenal gland. (**A**) Schematic of *RTL1* genomic locus and all SNP sites used in this study. Arrows indicate the direction of transcription. Shaded area indicates coding region on exon 4. Numbers indicate nucleotide positions on *RTL1* transcript. Lengths of sequences between exons are not drawn to scale. (**B**) Brain regions from a postmortem human fetal subject (−0.38 y/o): prefrontal cortex, cortex, thalamus, amygdala, hippocampus and cerebellum (top). Parent-of-origin-specific allelic expression of *RTL1* in different tissue was determined by Sanger sequencing of strand-specific cDNA. ‘M’ = maternal expression. ‘M^*^’ = maternally-biased expression (bottom). (**C**) Parent-of-origin-specific allelic expression of *RTL1* in the frontal cortex of a 3-year-old female was determined by Sanger sequencing. ‘M’ = maternal expression. ‘ND’ = non-detectable. (**D**) Parent-of-origin-specific allelic expression of *RTL1* in normal and tumor tissue from the temporal cortex of a 17-year-old female was determined by Sanger sequencing. ‘M’ = maternal expression. (**E**) Parent-of-origin-specific allelic expression of *RTL1* in the prefrontal cortex of a 29-year-old female was determined by Sanger sequencing. ‘M’ = maternal expression. (**F**) Parent-of-origin-specific allelic expression of *RTL1* in the prefrontal cortex of a 32-year-old female was determined by Sanger sequencing. ‘Bi’ = biallelic expression. (**G**) Comparison of expression levels of *RTL1* sense (top) and *RTL1* antisense (bottom) in postmortem prefrontal cortex from the 29-year-old and 32-year-old siblings from the same family quartet. Expression levels of *RTL1* were determined by qPCR and normalized to GAPDH. Student’s *t*-test, two-tailed, *n* = 3 per group. (**H**) Schematic diagram of the hypothalamic–pituitary–adrenal axis. Arrows indicate interactions between sites (left). Parent-of-origin-specific allelic expression of *RTL1* in the adrenal gland from the 45-year-old female (normal and tumor tissue) was determined by Sanger sequencing. ‘M’ = maternal expression. ‘Bi’ = biallelic expression (right). (**I**) Expression levels of *RTL1* sense (top) and *RTL1* antisense (bottom) in the normal and tumor tissue from the adrenal gland were determined qPCR. Expression levels were normalized to 18S rRNA. Student’s *t*-test, two-tailed, *n* = 3 per group. ^#^*P* < 0.1, ^*^^*^^*^P < 0.001. All data are shown in the mean ± SEM.

We next expanded the investigation of the imprinting status of *RTL1* to human brain with our available human postmortem and surgical resection brains of different ages and disease statuses. We found both *RTL1* sense and *RTL1* antisense were maternally, but not paternally expressed in all regions examined in 0.38 y/o fetal brains (prefrontal cortex, cerebral cortex, thalamus, amygdala, hippocampus and cerebellum) (Fig. 5B). Furthermore, examining samples from 3, 17, and 29-year-old individuals showed *RTL1* sense and *RTL1* antisense were both expressed maternally, but not paternally, in the cerebral cortex at three different stages of brain development (Fig. 5C–E). It is worth noting that there was an exception in one sample from a 32-year-old female with epilepsy, in which both *RTL1* sense and *RTL1* antisense were biallelically expressed in the prefrontal cortex (Fig. 5F). Moreover, levels of *Rtl1* sense and *Rtl1* antisense were up-regulated in the prefrontal cortex from the 32-year-old female compared with her 29-year-old sibling (Fig. 5G), possibly due to relaxation of imprinting of *RTL1* sense and *RTL1* antisense (Fig. 5F).

In addition to human brains, we examined *RTL1* imprinting status in the human adrenal gland due to the association of *RTL1* with anxiety (5), a psychological disorder in which the hypothalamic–pituitary–adrenal axis plays an integral role (Fig. 5H, left). Moreover, *Rtl1* was expressed in the adrenal gland of mice (Fig. 1A, and Supplementary Material, Fig. S1). We found that in normal adrenal gland tissue, *RTL1* sense and *RTL1* antisense were both expressed maternally, but not paternally (Fig. 5H, right). However, in adrenal gland tissue with an aldosterone-producing adenoma, *RTL1* sense showed biallelic expression (Fig. 5H, right). Levels of *RTL1* sense were unchanged but levels of *RTL1* antisense were downregulated in the adrenal gland containing this tumor (Fig. 5I). All imprinting patterns from human postmortem and surgical resection brains examined are summarized in Supplementary Material, Figure S12. Our results suggest the imprinting status of *RTL1* is maintained in human brain and adrenal gland.

### Paternal *Rtl1* knockout mice displayed a greater number of anxiety-like behaviors compared with controls

**Figure 6:**
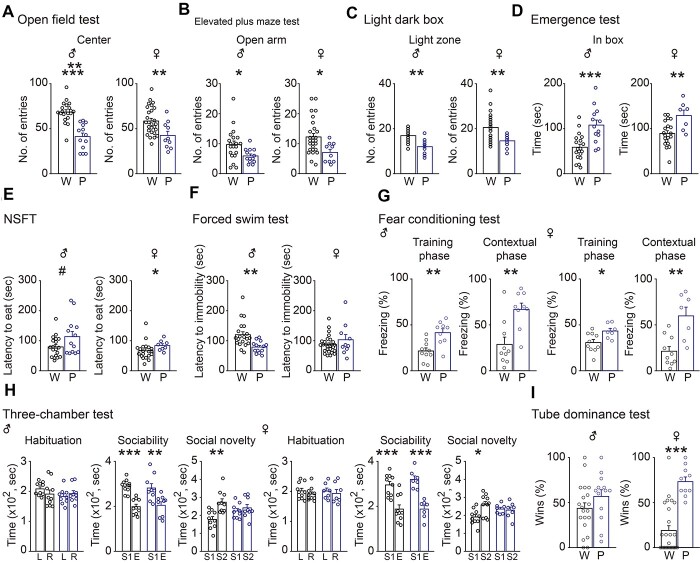
Paternal *Rtl1* knockout mice displayed more anxious, depressed, fear-related learning and memory, and social dominance behavior. Bar graphs showing differences in behaviors from male (left graphs) and female (right graphs) paternal *Rtl1* knockout mice and their corresponding wild-type controls. (**A**) Number of entries into the center in the open field test. (**B**) Number of entries into the open arm of the elevated plus maze test. (**C**) Number of entries into the light zone of the light–dark box test. (**D**) Amount of time spent ‘in box’ in the emergence test. (**E**) Latency to eat food in the center of the field in the novelty suppressed feeding test (NSFT). (**F**) Latency to immobility in the forced swim test. (**G**) Percent of time immobile (freezing) in the training (left), and contextual (right) phase of the fear conditioning test. (**H**) Three-chambered social test: Habituation (left) showing time spent in the left (L) and right (R) chamber; Sociability (middle) showing time spent in the chamber containing a stranger mouse (S1) and empty chamber (E); Social novelty (right) showing time spent in the chamber containing the familiar mouse (S1) and novel mouse (S2) (right). (**I**) Percentage of wins between wild-type mice (W) and paternal *Rtl1* knockout mice (P) in the tube dominance test. Student’s *t*-test, two-tailed, Mann–Whitney rank sum test, or one-way ANOVA with Holm-Sidak *post hoc* comparison, ^#^*P* < 0.1, ^*^P < 0.05, ^*^^*^P < 0.01, ^*^^*^^*^P < 0.001, ^*^^*^^*^^*^^*^P < 0.00001. W (male), *n* = 9–20; P (male), *n* = 9–13; W (female), *n* = 10–27; P (female), *n* = 7–10. All data are the mean ± SEM.

The association of *RTL1* with anxiety-like behaviors (5) suggests loss of RTL1 could contribute to anxiety-like or other emotion-related behaviors. Therefore, we first performed tests with five different behavioral paradigms related to anxiety-like behaviors. The open field test showed, compared with WT controls, *Rtl1^m+/p-^* mice made fewer entries into the center of the field (Fig. 6A). *Rtl1^m+/p-^* mice also made fewer entries into the open arm with the elevated plus maze test (Fig. 6B), and the light zone with the light–dark box test (Fig. 6C). The emergence test showed, compared with WT controls, *Rtl1^m+/p-^* mice were ‘in box’ of the field for a greater number of seconds (Fig. 6D). Lastly, the novelty-suppressing feeding test demonstrated *Rtl1^m+/p-^* mice in a condition of hunger had a greater latency to eat when food was placed in the center of the field compared with WT controls (Fig. 6E). Taken together, our results suggested a causal relationship between loss of *Rtl1* and anxiety-like behaviors.

### Paternal *Rtl1* knockout mice displayed more depressive, fear-related and social dominance behaviors compared with controls

To further investigate whether loss of *Rtl1* could contribute to other emotion-related behaviors, we performed tests related to depressive-like responses and fear-related learning and memory behaviors. The forced swim test, which measures immobility as a depressive-like response, demonstrated male *Rtl1^m+/p-^* mice showed a decrease in latency to immobility, indicating depressive-like behavior (Fig. 6F). The contextual fear conditioning test, which uses immobility (freezing) in response to a stimulus as a measure for fear-related learning and memory, showed that compared with WT controls, male and female *Rtl1^m+/p-^* mice exhibited a higher percentage of time in a freezing state during the conditioning contextual phase (Fig. 6G), suggesting *Rtl1^m+/p-^* mice have a greater level of fear-related learning and memory behaviors. Taken together, our results suggest loss of *Rtl1* contributes to depressive-like and fear-related learning and memory behaviors.

Dysfunctional anxiety-like and emotion-related behaviors could impact social behaviors. Therefore, we used two testing paradigms to evaluate if *Rtl1^m+/p-^* mice exhibited atypical social behaviors. The three-chambered test, which measures sociability and novelty, demonstrated *Rtl1^m+/p-^* mice responded appropriately by spending more time in a chamber with a stranger mouse than an empty chamber, indicating normal sociability (Fig. 6H, middle), but had deficits in social novelty (Fig. 6H, right). The tube dominance test demonstrated female *Rtl1^m+/p-^* mice were significantly more socially dominant than their WT controls, whereas social dominance did not differ between male *Rtl1^m+/p-^* and WT control mice (Fig. 6I). Our results provide additional evidence suggesting loss of *Rtl1* contributes to atypical social behaviors.

### Paternal *Rtl1* knockout mice displayed hypoactivity and weak muscle strength

**Figure 7:**
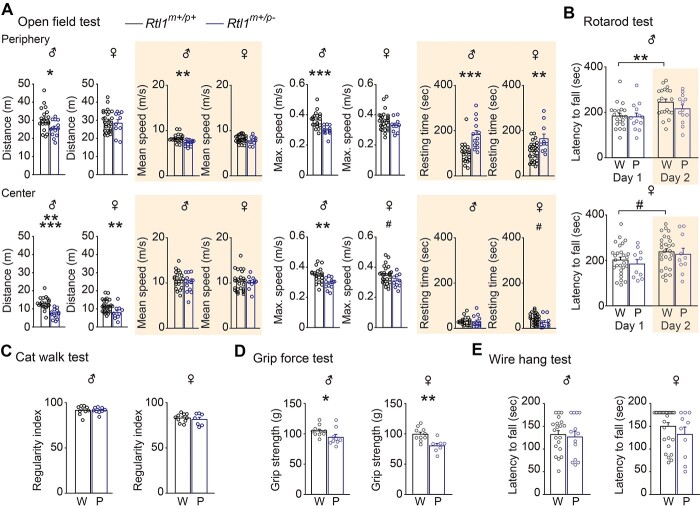
Paternal *Rtl1* knockout mice showed hypoactivity and decreased muscle strength. Bar graphs showing differences in behaviors from male (left graphs) and female (right graphs) paternal *Rtl1* knockout mice and their corresponding wild-type controls. (**A**) Open-field test indicating total distance travelled in the periphery and center of the field (panel 1). Bar graph showing mean speed in the periphery and center of the field (panel 2). Bar graph showing maximal speed in the periphery and center of the field (panel 3). Bar graph indicating total resting time in the periphery and center of the field (panel 4). (**B**) Latency to fall at day 1 and day 2 in the rotarod test. (**C**) Bar graph indicating regularity index in the catwalk test. (**D**) Grip strength in the grip force test. (**E**) Latency to fall in the wire hang test. Bar graphs are labeled W and P indicated wild-type mice and paternal *Rtl1* knockout mice, respectively. Student’s *t*-test, two-tailed, Mann–Whitney Rank Sum test, or Two-way repeated measured ANOVA with Holm-Sidak *post hoc* comparison, ^#^*P* < 0.1, ^*^P < 0.05, ^*^^*^P < 0.01, ^*^^*^^*^P < 0.001, ^*^^*^^*^^*^^*^P < 0.0001. W (male), *n* = 9–20; P (male), *n* = 9–13; W (female), *n* = 10–27; P (female), *n* = 7–10. All data are the mean ± SEM.

*Rtl1* plays an important role in fetal/neonatal skeletal muscle development (10). To investigate whether loss of *Rtl1* could contribute to defects in locomotor activity, we performed the open field test with *Rtl1^m+/p-^* mice and their corresponding controls (Fig. 7A). We observed that *Rtl1^m+/p-^* mice displayed a decrease in distance travelled, mean speed, maximal speed and increased resting time (Fig. 7A). We also evaluated motor coordination, muscle strength and endurance to further investigate whether loss of *Rtl1* could contribute to the deficits of motor activity. The rotarod test showed *Rtl1^m+/p-^* mice had normal motor coordination, but there were deficits in motor learning (Fig. 7B). The regularity index on the catwalk test did not differ between *Rtl1^m+/p-^* mice and WT controls (Fig. 7C). The grip force test indicated *Rtl1^m+/p-^* mice had lower muscle strength compared with WT controls (Fig. 7D), but endurance, as measured by the wire hang test, did not differ (Fig. 7E). Because we observed deficits of motor learning in *Rtl1^m+/p-^* mice, we further evaluated whether there were deficits in learning and memory with the T-maze rewarded alternation test (Supplementary Material, Fig. S13A) and novel object recognition test (Supplementary Material, Fig. S13B). *Rtl1^m+/p-^* mice displayed normal short-term and long-term memory (Supplementary Material, Fig. S13). Furthermore, the T-maze spontaneous alternation test (Supplementary Material, Fig. S14A) indicated *Rtl1^m+/p-^* mice had no deficits in exploratory behaviors or repetitive/stereotypical behaviors; the hot plate test demonstrated *Rtl1^m+/p-^* mice had no deficits in nociception (Supplementary Material, Fig. S14B). Our results showed *Rtl1^m+/p-^* mice had lower locomotor activity and weaker muscle strength.

### LC neurons from paternal *Rtl1* knockout mice demonstrated increased delays in time of onset for action potentials and inward currents and decreased neuronal excitability

The LC is a nucleus in the pons of the brain stem, which is involved with physiological responses to stress and panic. The basolateral complex of the amygdala receives a dense noradrenergic innervation from the LC, and norepinephrine (NE) levels increase in the basolateral amygdala (BLA) with exposure to stress stimuli (11). Neurons in the LC are activated by stressful stimuli (12) and mediate the stress-induced enhancement of NE neurotransmission in the BLA (13). Noradrenergic projections from the LC to the BLA promote anxiety-like behavior (14).

**Figure 8:**
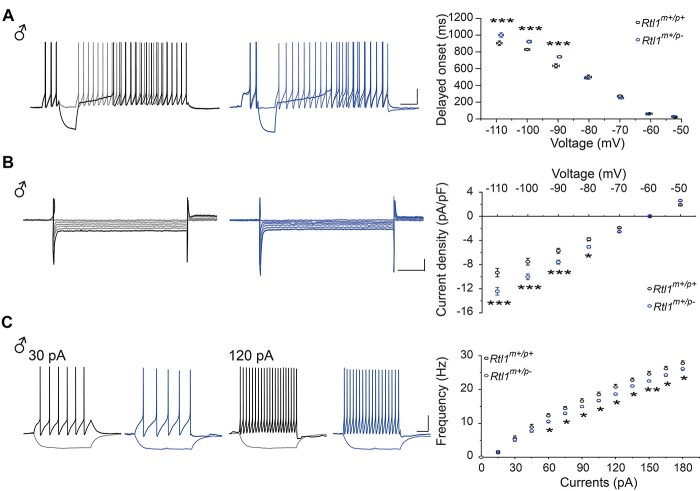
LC neurons from paternal *Rtl1* knockout mice displayed increased delays in onset time of action potentials and inward currents and decreased neuronal excitability. (**A**, left) Representative traces of current clamp recordings from LC neurons of male *Rtl1^m+/p+^* and *Rtl1^m+/p-^* mice demonstrating the hyperpolarization of membrane voltage prolongs the ‘onset’ of action potentials. Black traces represent *Rtl1^m+/p+^* mice; blue traces represent *Rtl1^m+/p-^* mice. The dark line represents depolarization at −60 mV and the light line represents depolarization at −110 mV. Scale bar represents 20 mV/300 ms. (A, right) The relationship between delay time and membrane voltage (*V*_m_) show the delayed excitation of LC neurons is in a *V*_m_-dependent manner. (**B**, left) Representative traces evoked from LC neurons of male *Rtl1^m+/p+^* and *Rtl1^m+/p-^* mice in response to voltage steps from −50 to −110 mV. Scale bar represents 150 pA/100 ms. (B, right) The current–voltage relationships show the linear current responses to hyperpolarizing voltage from LC neurons. (**C**, left) Representative response to different current injections of the LC neurons from male *Rtl1^m+/p+^*, and *Rtl1^m+/p-^* mice. Scale bar represents 20 mV/200 ms. (C, right) Quantification of frequency of action potential firing during indicated magnitude of current injection. Two-way repeated measures ANOVA with Holm-Sidak *post hoc* comparison, ^*^P < 0.05, ^*^^*^P < 0.01, ^*^^*^^*^P < 0.001. *Rtl1^m+/p+^* mice, *n* = 12 slices from 7 male mice; *Rtl1^m+/p-^* mice, *n* = 13 slices from 7 male mice. All data are the mean ± SEM.

To investigate whether the electrophysiological properties of LC neurons in *Rtl1^m+/p-^* mice could explain any difference that might affect the downstream behavioral phenotypes we observed, we performed whole-cell patching on LC neurons of *Rtl1^m+/p-^* mice. First, to determine if electrophysiological properties of neurons recorded from male and female WT mice differed from *Rtl1^m+/p-^* mice, spontaneous firing activity of LC neurons was recorded for 1 min, and template search was used for spontaneous firing rate analyses. The values of the firing rate of LC neurons were similar between WT and *Rtl1^m+/p-^* mice (Supplementary Material, Fig. S15). Moreover, there were no differences in input resistance, membrane time constants and capacitances between WT and *Rtl1^m+/p-^* mice (Supplementary Material, Fig. S15). Second, we examined the specific electrical pattern of LC neurons of male WT and *Rtl1^m+/p-^* mice, and confirmed hyperpolarization of membrane voltage prolonged the ‘onset’ of action potentials (Fig. 8A) and the linear current responded to hyperpolarizing voltage (Fig. 8B). LC neurons from *Rtl1^m+/p-^* mice showed an increase in both delayed onset time of action potentials and inward currents compared with neurons from WT mice (Fig. 8A and B). To further examine changes in excitability of LC neurons of male WT and *Rtl1^m+/p-^* mice, the evoked neuronal firing activity was also evaluated by injection of depolarizing current steps. Compared with LC neurons from WT mice, the frequency was reduced after the depolarization current step (Fig. 8C). Moreover, analysis of the action potential features showed there was a decrease in the maximum rise slope and the maximum decay slope in the first action potentials of LC neurons from *Rtl1^m+/p-^* mice compared to LC neurons from WT mice (Supplementary Material, Fig. S16). In addition, half-width, an index of action potential duration, was increased in neurons from *Rtl1^m+/p-^* mice (Supplementary Material, Fig. S16). However, there was no significant difference in resting membrane potential (RMP), threshold, the first action potential amplitudes, time to peak of first action potentials, and the after-hyperpolarization potential (AHP) amplitudes between WT and *Rtl1^m+/p-^* mice (Supplementary Material, Fig. S16). Specific electrophysiological properties and excitability of LC neurons from female *Rtl1^m+/p-^* mice (Supplementary Material, Figs S16 and S17) were consistent with LC neurons from male *Rtl1^m+/p-^* mice. Taken together, our results showed that loss of *Rtl1* affected LC neuron properties.

## Discussion

Our study of *Rtl1* knockout mice provides a comprehensive overview of the imprinting patterns of *Rtl1* in mouse brains. Our examination of human postmortem and surgical resection brain tissues provides complementary information of the imprinting patterns of *RTL1* in human brain. The increased anxiety-like behaviors and emotion-related behaviors of depression and fear-related learning and memory in *Rtl1^m+/p-^* mice provide support for the role of dysfunctional RTL1 as a contributor to anxiety. Compared with WT mice, *Rtl1^m+/p-^* mice also showed no social novelty behavior, greater social dominance and hypoactivity. Our findings for the neuronal properties of the LC in *Rtl1^m+/p-^* mice might provide a potential link between neuronal mechanisms of RTL1 and emotion-related behavioral phenotypes. Our results indicate the key features of *RTL1*-related human brain disorders are recapitulated in *Rtl1^m+/p-^* mice, and this mutant mouse could be a powerful model for understanding relevant human brain disorders with an emphasis on emotion- and social-related phenotypes.

Our results showed RTL1 is predominantly expressed in the brain and adrenal gland among the postnatal organs in mice. Adrenal glands produce hormones that respond to stress. Our findings showed loss of *Rtl1* contributes to emotion-related dysfunctions with more robust impacts on anxiety-like behaviors. Moreover, *RTL1* has been linked to broad anxiety disorders (5). Location of RTL1 expression in both mouse and human adrenal gland demonstrated a potential link between the function of RTL1 and stress-related phenotypes. Moreover, higher expression of RTL1 in embryonic and neonatal mouse brains imply a potential role for RTL1 in early brain development. Indeed, we observed decreased brain weight in our homozygous *Rtl1* knockout mice. Moreover, decreased weight of the placenta at E16.5 and decreased stomach weight at P0 both contributed to decreased body weight at postnatal stages in *Rtl1^m+/p-^* mice. Decreased stomach weight at P0 due to impaired suckling behavior could explain low survival rate in neonatal *Rtl1^m+/p-^* mice.

Although five *Rtl1* transcript isoforms encode the same RTL1 protein, their expression patterns were different between placenta and brain. *Rtl1 Ex1a* was predominantly expressed in placenta, and *Rtl1 Ex1b* was predominantly expressed in brain. The physiological purposes in the use of two different *Rtl1* isoforms in two different organs are not clear, despite producing the same RTL1 protein. Even in the brain, *Rtl1 Ex1d,* rather than *Ex1b,* was predominantly expressed in the cerebellum. How the expression of *Rtl1* transcript isoforms is regulated in different organs and brain regions and functional consequences of different transcript isoforms for the same protein need to be further investigated.

There are seven microRNAs within the coding exon of *Rtl1*; therefore, we created an indel mutation within the coding exon of *Rtl1* to avoid deleting both *Rtl1* and surrounding microRNAs simultaneously. With such specificity of *Rtl1* deletion, the results from our *Rtl1* knockout mice provide a clear and specific causal relationship between loss of RTL1 and its corresponding phenotypes. Moreover, due to this limitation, we could not insert *loxP* sites without disrupting microRNAs within the coding exon of *Rtl1*. Therefore, creating an *Rtl1* conditional knockout mice is not feasible. Such technical hurdles limit our ability to explore the cell-type-specific contribution of RTL1 to brain functions (15).

*Rtl1* was expressed paternally, but not maternally, in brain stem, thalamus and hypothalamus, which is consistent with our previous finding (9). *Rtl1* was also expressed in amygdala-related regions such as the BNST, AHiPM and APir; however, its imprinting status was relaxed in the LSD. It would be of interest to investigate further why the LSD tends to be resistant to being fully imprinted. Although there was no detectable maternally expressed RTL1 protein in the AHiPM, APir or PB, we observed fewer RTL1-expressing cells in those three brains regions of *Rtl1^m−/p+^* mice. It would be of interest for future studies to examine how loss of maternally-expressed *Rtl1* transcript in AHiPM, APir or PB contributes to fewer RTL1-expressing cells. Two possibilities must first be examined; whether there is decreased viability of RTL1-expressing cells or decreased levels of paternally-expressed *Rtl1* transcript. Although previous findings showed *Rtl1* sense is expressed maternally, but not paternally, in olfactory bulb, cerebral cortex, and cerebellum of mice (9), we did not observe RTL1 protein in these three brain regions, which suggests levels of maternally expressed *Rtl1* sense were too low to detect its protein. Moreover, the results of our human postmortem cerebral cortex also showed *RTL1* sense was maternally, but not paternally expressed, which is consistent with our previous expression results of *Rtl1* sense in mouse cerebral cortex. The one conclusion we can draw is that the imprinting status of *RTL1* is conserved in both human and mouse cerebral cortex.

A previous study showed *Rtl1* antisense regulates *Rtl1* sense in *trans* by guiding RISC-mediated cleavage of its mRNA (16). Although currently there is no direct evidence to demonstrate whether *Rtl1* antisense regulates *Rtl1* sense in *cis*, our findings in human cerebral cortex imply it is unlikely or may not fully occur. We also observed a relaxation of imprinting status of *RTL1*, which also corresponded with an increased expression level. Although there was no change in imprinting status of *RTL1* antisense in normal and tumor adrenal gland, we observed a decrease in *RTL1* antisense in the adrenal gland containing tumor. These differences could be due to changes in levels of miRNAs within *RTL1* antisense in the adrenal gland containing tumor. Our results revealed the imprinting status of *RTL1* is maintained in the human brain. Contrary to the paternal expression pattern found in mice, *RTL1* in subcortical regions such as the thalamus, amygdala and hippocampus were all found to be maternally, but not paternally expressed in the fetal brain. Due to the scarcity of available human brain tissue, we did not examine the brain stem, another brain region in which *RTL1* was expressed paternally, but not maternally, and abundantly in mice. Further investigations could address this issue by examining samples of brain regions other than the cortex, and preferably including samples obtained from tissues at different developmental stages. Another finding of note is that *RTL1* was expressed paternally, but not maternally, in human induced pluripotent stem cells (hiPSCs)-derived cortical neurons (Supplementary Material, Fig. S18). The discrepancy of the *RTL1* imprinting status between hiPSCs-derived cortical neurons and human postmortem or surgical resection cortex needs to be investigated further. The discrepancy may have resulted from differences in developmental stages or dysregulation of imprinting during generation of hiPSCs and derivation of hiPSCs into cortical neurons, but these are speculations and should be further examined.

Although five *Rtl1* isoforms translated into the same RTL1 protein, we only observed two RTL1 protein bands. However, several issues need to be clarified. First, we confirmed the two protein bands belong to RTL1 protein, which is indicated by the disappearance of two protein bands from embryonic brains and placenta (Fig. 1I, left) and postnatal brains and adrenal glands (Fig. 1I, right) of *Rtl1* homozygous knockout mice compared with their wild-type controls. Second, because five *Rtl1* gene isoforms were translated into the same RTL1 protein (Fig. 1C), we suspect that one of the two RTL1 proteins with a higher molecular weight could be due to post-translational modifications.

Anxiety disorders, including generalized anxiety disorder, social anxiety disorder, separation anxiety disorder, panic disorder, specific phobias, and selective mutism, are the most prevalent mental disorders (17,18). Approximately 34% of the population is affected by an anxiety disorder at some time during their lifetime (18). Neuronal circuits for fear and anxiety have been identified due to the advancement of genetic tools and imaging techniques (19). Key brain regions that are involved in fear and anxiety include the amygdala, the medial prefrontal cortex (mPFC) and the hippocampus. Other brain regions such as LC, raphe nuclei, periaqueductal grey, parabrachial nucleus, lateral septum, hypothalamus, ventral tegmental area, bed nucleus of the stria terminalis are also included in these circuits. Both local microcircuits and long-range projection-specific pathways have helped to advance our view of how the brain produces fear and anxiety states and the resulting adaptive defensive behaviors.

The hypothalamus-pituitary–adrenal axis (HPA axis) is a complex set of direct influences and feedback interactions among three endocrine glands, which controls reactions to stress and regulates many body processes including mood and emotions. The HPA axis is involved in the neurobiology of mood disorders and functional illness such as anxiety disorders. Our behavior results solidify the causal relationship between loss of *Rtl1* and emotion-related behaviors such as anxiety, depression, and fear-related learning and memory. In our other behavioral assays, *Rtl1^m+/p-^* mice lacked social novelty and displayed an increase in social dominance. Hypoactivity and decreased muscle strength were observed in *Rtl1^m+/p-^* mice, which could be due to impaired muscle development (10). Our immunofluorescence data demonstrated high levels of expression of *Rtl1* in the LC and LC neurons from *Rtl1^m+/p-^* mice, while our electrophysiological data indicated decreased neuronal excitability, increased delayed time of onset, and increased inward currents. However, it is premature to draw a link from our electrophysiological results in LC neurons to our behavioral findings in *Rtl1^m+/p-^* mice. The lack of any observable difference of foot nociception between *Rtl1^m+/p-^* and wild-type mice (Supplementary Material, Fig. S14B) caused us to eliminate the possibility that the sensitivity of the foot shot might contribute to the difference in freezing time in the training phase of fear conditioning test between *Rtl1^m+/p-^* and wild-type mice.

Our findings provide evidence that the LC might be a starting point for future studies examining the role of RTL1 in anxiety-like and emotion-related behaviors. The anxiety and emotion-related phenotypes observed in *Rtl1^m+/p-^* mice could explain the social-related phenotypes observed in *Rtl1^m+/p-^* mice. However, future studies are needed to investigate detailed circuit and molecular mechanisms of RTL1 in emotion-related phenotypes, as these may serve as targets of therapy for individuals with *RTL1*-linked brain disorders.

## Materials and Methods

### Western blotting

Tissue was homogenized in 1× RIPA lysis buffer [150 mm NaCl, 50 mm Tris/HCl, 5 mm EDTA, 0.5% sodium deoxycholate, 1% Nonidet P-40, 0.1% SDS) with a protease inhibitor cocktail (Hycell, HC100-007)]. Following centrifugation, the supernatant was collected, and protein concentration was measured with the Pierce BCA Protein Assay Kit (Thermo Scientific, 23227). Total protein lysates (30 μg) were separated by 6% (stacking layer)/10% (separating layer) SDS-PAGE and transferred to nitrocellulose membranes. Immunoblotting was performed using the following primary antibodies: rabbit anti-RTL1 (1:1000, Dr Stewart’s lab, YZ 2843 (2), and Genetex, PN 90749), mouse anti-beta-ACTIN (1:3000, Sigma, A1978), rabbit anti-GAPDH (1:6000, Genetex, GTX110118) and rabbit anti-alpha-TUBULIN (1:1000, Cell signaling, 2144). Primary antibodies were detected with their corresponding secondary antibodies: IRDye680 donkey anti-mouse IgG (H + L) (1:15 000, LI-COR, 926-68072) and IRDye800CW donkey anti-rabbit IgG (H + L) (1:15 000, Li-COR, 926-32213). Protein bands were visualized with an Odyssey^®^ Fc Dual-Mode Imaging System (LI-COR Biosciences). To control for protein loading, each protein level was normalized to ACTIN, GAPDH or TUBULIN levels detected in each sample. The anti-RTL1 epitope (226 a.a.) resides at the N-terminus (amino acid 89–314 of NP_908998.1) of RTL1 (1744 a.a.) (2).

### Reverse transcription quantitative PCR analysis

Total RNA was extracted from mouse brains, human postmortem prefrontal cortex or human surgical resection adrenal gland tissues using RNeasy^®^ Lipid Tissue Mini Kit (QIAGEN, 74804). For human samples, RNAs were treated by RNase-Free DNase (QIAGEN, 79254), then reverse-transcribed by SuperScript^™^ III Reverse Transcriptase kit (Invitrogen, 18080044) into *RTL1* sense or antisense cDNA, respectively, with strand-specific primers (primer name: RTL1_Q, sequences listed in Supplementary Material, Table S1). Prior to quantitative PCR (qPCR) analysis, the presence of genomic DNA in the samples was checked. qPCR was conducted with a StepOnePlus Real-Time PCR System (Applied Biosystems) with *Power* SYBR^™^ Green PCR Master Mix (Applied Biosystems, 4367659). For mouse samples, qPCR analysis was conducted with Power SYBR Green RNA-to-CT 1-Step Kit (Applied Biosystem, 4389986) and StepOnePlus Real-Time PCR Systems (Applied Biosystem). Primer sequences of five *Rtl1* isoforms were described in the previous study (2). Samples were run in triplicate, and relative expression values were determined by the comparative Ct method (2-ΔΔCt). *Tbp*, *GAPDH* and *18S rRNA* were used as reference genes for the mouse brains, human prefrontal cortex and human adrenal gland groups, respectively.

#### Mice

*Rtl1* knockout mice on a C57BL/6J background were generated by introducing an indel mutation (Fig. 1F) in exon 2 using CRISPR/Cas9 method to avoid destroying the intrinsic miRNA regions (Fig. 1F). *Rtl1* sgRNA is 5′-GCTCTTGCATTTCCTTGAGG-3′. *Rtl1* 3XSTOP ssODN is AGGTTGAGGCCCAGGCGGGCTCAGATAGTGGCCCAGCCCAGGAAGAAAAGGAGCCACCCAG*CT*G*A*CCCCTC*T*AG*T*AAATGCAAGAGCTGCCCACTAATCTACTCCAAGAAGTGGAGGAGCCATCCAGTGGCCCCCACCAG. Mice were group-housed in ventilated cages on a 12-h light/dark cycle (lights off at 8 p.m.) with *ad libitum* access to food (PicoLab^®^ Rodent Diet 20, 5053) and water. Male *Rtl1* heterozygous knockout mice were mated to female C57/BL6J mice to obtain *Rtl1*^m+/p-^ mice. Female *Rtl1* heterozygous knockout mice (HET) were mated to male C57/BL6J mice to obtain *Rtl1*^m−/p+^ mice. The National Taiwan University College of Medicine and the College of Public Health Institutional Animal Care and Use Committee (IACUC) approved all procedures (IACUC no.: 20170082). All experiments were performed in accordance with the approved guidelines.

### Genotyping

PCR for genotyping and later Sanger sequencing was performed with primer sequences *ZF* (5′-CTT CAG AGC ACA CTC CTT TC-3′) and *ZR* (5′-ACT TCC CGG AGT AGA TCA GT-3′). PCR for TA cloning was performed with primer sequences *ZF* (5′-CTT CAG AGC ACA CTC CTT TC-3′) and *500R* (5′-TCA CAG GGG TCG CTG GAT-3′). PCR for regular genotyping by PCR was performed with primer sequences *WT* (5′-CCA GTG GCC CCC TCA AG-3′) and *Homo* (5′-GCT CTT GCA TTT CCT TGG-3′). The PCR cycling conditions for both *ZF* and *ZR* primers and *ZF* and *500R* primers were as follow: 95°C for 30 s, followed by 35 cycles of 95°C for 15 s, and 60°C for 30 s and then hold at 12°C The size of PCR product was 487 bp for *ZF* and *500R* primers, and 789 bp for *ZF* and *500R* primers, PCR cycling conditions for *WT* and *500R* primers and *ZF* and *Homo* primers were as follows: 40 cycles at 95°C for 15 s, and 63°C for 30 s, and then hold at 12°C. Amplification was performed on a C1000 Touch Thermal Cycling (Bio-Rad Laboratories). For TA cloning, electrophoresis was followed by gel purification. Purified PCR products were subcloned by TOPO TA cloning kits for sequencing (Invitrogen, 250030), and the ligation product was utilized in transformation into One Shot TOP10 Chemically Competent *Escherichia coli* (Invitrogen, K457510) or ECOS^™^ X competent *Escherichia coli* (Yeastern Biotech, YE610-10). Plasmids were isolated from selected clones by Presto Mini Plasmid kit (Geneaid, PHD100) and were sent to Genomics Biosci & Tech company for Sanger sequencing.

### Immunofluorescence staining

P49 mouse brains were immersion-fixed with 4% paraformaldehyde in 0.1 M phosphate buffer (PB) (pH 7.4) for 12 h and then cryoprotected with 30% sucrose in 0.1 M PB at 4°C overnight. Coronal sections (50 μm) were cut with a cryostat (Leica, CM3050 S) and collected in phosphate buffered saline (PBS), incubated with 0.3% Triton X-100 in 1× PBS for 30 mins, and blocked in 1× PBS containing 5% goat serum and 0.3% Triton X-100 for 1 h. Sections were further incubated at 4°C overnight with anti-RTL1 antibody (1:2000, Gentex), anti-TH antibody (1:500, Millipore, MAB318) or anti-NeuN antibody (1:500, Millipore, MAB377), followed by incubation with the appropriate secondary antibody for 2 h at room temperature: Alexa Fluor 594 goat anti-mouse IgG (H + L) antibody (1:500, Invitrogen, A32742 for TH; 1:500, Jackson ImmunoResearch, 115-585-003 for NeuN); Alexa Fluor 488 goat anti-rabbit IgG (H + L) antibody (1:500, Invitrogen, A32731 for RTL1). Tissues were all labeled with DAPI (1:10 000, Invitrogen, D-1306). Sections were washed in 1× PBS containing 0.1% Triton X-100 three times (5 min/wash) following incubations with primary and secondary antibodies. Sections were cover slipped with Fluoromount aqueous mounting medium (Sigma, F4680) and images were acquired using a Zeiss LSM880 confocal microscope. Montage images were acquired with a 20×/0.8 NA objective and enlarged images were acquired with a 63×/1.4 NA objective. For defining brain regions and nuclei of interest, overlapping photos of coronal brain sections were guided from the Allen Mouse Brain Atlas.

### *RTL1* imprinting status in human tissue

To identify *RTL1* imprinting status in human brain tissue obtained through surgical resection or postmortem, we studied three family trios, one family quartet and one family duet. The first family trio was comprised of the parents and postmortem fetal tissue (female). The second family was comprised of the parents and surgical resection tissue from their 3-year-old daughter. The third was comprised of the parents and surgical resection tissue from their 17-year-old daughter. The family quartet was comprised of the parents and postmortem tissue from two children, who were sisters, aged 29 and 32 years (20). Samples from the family duet were comprised of a mother and surgical resection tissue from her daughter, aged 45 years. In addition, an additional family trio was studied to identify *RTL1* imprinting status in hiPSC-derived cortical neurons. The family trio was comprised of the parents and one offspring, aged 16 years.

We examined post-mortem fetal tissue from the prefrontal cortex, cortex, thalamus, hippocampus, amygdala and cerebellum and parental blood of the first family trio. We examined surgical resection tissue from the frontal cortex of the 3-year-old and parental blood from the second family trio. Surgical resection samples from the 17-year-old brain included normal and tumor tissues from the temporal cortex and parental blood. Postmortem tissue from prefrontal cortex (Brodmann area 8) of the 29-year-old and postmortem tissue from the prefrontal cortex (Brodmann area 10) of the 32 year old, and parental blood was assessed from the family quartet. We examined surgical resection samples of normal and tumor tissues from the adrenal gland of the 45 year old and the mother’s blood of the family duet. The tissues examined were based on availability. The assessed hiPSC-derived cortical neurons were generated using blood samples from the offspring and parents in the last family trio. More detailed information of the families and samples used are shown in Supplementary Material, Table S2.

The institutional review boards (IRBs) of the participating institutions approved all experimental protocols. All experiments were carried out in accordance with the approved guidelines of the IRBs of the participating institutions. Written informed consent was obtained from both parents. Human quartet samples were obtained from the University of Utah Autism Research Program. The IRBs of the University of Utah, Icahn School of Medicine and National Taiwan University approved the analyses of samples and data in this study. Human trios and duet samples (including the family trio in the hiPSC study) were obtained from National Taiwan University Hospital. The IRBs of the National Taiwan University approved the analyses of samples and data in this study (IRB numbers: 201308050RINB for human tissue study and 201901027RIND for hiPSC study). All postmortem and surgical resection tissue was fresh-frozen, except for temporal cortex tissue from the 17 year old, which was formalin-fixed and paraffin-embedded. All tissue was stored at −80°.

### hiPSC-derived cortical neuron preparation

Whole blood samples were obtained from a 16-year-old male Taiwanese patient diagnosed with autism. Density gradient centrifugation was then performed, with Ficoll-Paque^™^ PLUS Media (Cytiva, 17144003) as the gradient solution to isolate peripheral blood mononuclear cells (PBMCs). The isolated PBMCs were reprogrammed with CytoTune^™^-iPS 2.0 Sendai Reprogramming Kit (Invitrogen, A16517), following the feeder-dependent protocol, and induced pluripotent stem cells (iPSCs) were generated. Next, the hiPSCs underwent neural induction for 12 days, forming neural stem cells (NSCs), which were expanded and differentiated into cortical neurons. The above steps were performed following the protocol described by Shi *et al.* (21). The hiPSC-derived cortical neurons were collected for allele-specific expression analysis at differentiation day 35.

### SNP site selection

SNP sites used in identification of *RTL1* imprinting status were identified by searching genomic DNAs of the offspring. Blood genomic DNA was extracted using NucleoSpin^®^ Blood kit (Macherey-Nagel, 740951), or extracted from fresh-frozen brain tissue using NucleoSpin^®^ Tissue kit (Macherey-Nagel, 740952). Sequences of *RTL1* were then amplified with specific primers (primer sequences are listed in Supplementary Material, Table S1) and Taq polymerase (GoTaq^®^ Master Mixes (Promega, M7122); or AptaTaq^™^ Fast DNA Polymerase (Roche, 6879110001) for some of the samples), in C1000 Touch Thermal Cycler (Bio-Rad Laboratories). The PCR cycling condition was as follows: 95°C for 30 s followed by 41 cycles of 95°C for 15 s and 60°C for 30 s. The PCR product was purified, and Sanger sequencing was performed at Genomics Biosci & Tech company. SNP sites were chosen to be used for identification of *RTL1* imprinting status if parents and offspring had heterozygous alleles, and each allele’s parent of origin could be inferred from Sanger sequencing results. Details of these SNP sites are shown in Supplementary Material, Table S3 and Supplementary Material, Figure S19.

### *RTL1* sense and antisense preparation

Depending on the status of tissue samples (fresh or formalin-fixed paraffin-embedded), total RNA was extracted from human postmortem or surgical resection tissue by RNeasy^®^ Lipid Tissue Mini Kit (QIAGEN, 74804) or NucleoSpin^®^ FFPE RNA kit (Macherey-Nagel, 740969). RNAs were treated by RNase-Free DNase (QIAGEN, 79254), then reverse-transcribed by SuperScript^™^ III Reverse Transcriptase kit (Invitrogen, 18080044), or by High-Capacity cDNA Reverse Transcription Kit (Applied Biosystems, 4368814) for hiPSC samples, into *RTL1* sense or antisense cDNA, respectively, with strand-specific primers. For cDNA prepared for allele-specific expression analysis, strand-specific primers were designed to transcribe sequences containing SNP sites of interest found in the SNP site selection step.

#### Allele-specific expression analysis

Parts of the *RTL1* sequence with SNP sites of interest were amplified by PCR. PCR amplification was performed with specific primers and Taq polymerase [Platinum^™^*Taq* DNA Polymerase (Invitrogen, 10966018); or GoTaq^®^ Master Mixes (Promega, M7122); or AptaTaq^™^ Fast DNA Polymerase (Roche, 6879110001)], in C1000 Touch Thermal Cycler (Bio-Rad Laboratories). PCR cycling conditions varied depending on specific primers used, cDNA concentration and sample quality (cycling conditions are listed in Supplementary Material, Table S1). PCR product was purified and Sanger sequencing was performed at Genomics Biosci & Tech company. Imprinting status of *RTL1* sense and antisense was interpreted based on expressed alleles shown in Sanger sequencing results and these alleles’ parent of origin. If biallelic expression was indicated, the presence of genomic DNA in cDNA samples was confirmed by PCR amplification with primers annealing to introns of GAPDH [primer name: GAPDH_gDNA, sequences and PCR cycling conditions shown in Supplementary Material, Table S1) and Taq polymerase (2× SuperRed PCR Master Mix (TOOLS, TE-SR01)] to rule out the possibility of genomic DNA contamination.

### Behavioral measures

All behavioral experiments were performed with two different groups of male mice and female mice. The male group was comprised of mice derived from seven litters of WT mice (*n* = 20), and four litters of *Rtl1^m+/p-^* mice (*n* = 13). The female group was comprised of mice derived from 13 litters of WT mice (*n* = 27), and four litters of *Rtl1^m+/p-^* mice (*n* = 10). One to two weeks prior to behavior tests, mice were handled 2–3 times per week, 1–2 min each day, to familiarize the mice with the experimenter and to reduce anxiety. Body weight was measured every 7 days before the tests and during the tests period and as a means of monitoring the health of the mice. Behavior tests began when male and female mice were 13–14 weeks of age and were completed within 3–4 months with adequate time intervals between the individual tests for the mice to recover. All test mice were housed with the same conditions during the test period. To avoid of the confounding factors from stress and fatigue for mice during multiple behavioral tests, the behavior tests were conducted in sequential order from the least to most stressful (Supplementary Material, Table S4) with a gap of 2–10 days between each test, depending on the stress level of the test. The habituation time in the test room for mice was at least 30 min in most of the tests, and at least 1 h habituation in the anxiety-related experiments. All behavior tests were conducted during the dark cycle (9 a.m.–7 p.m.); anxiety-related experiments were conducted from 12 p.m.–7 p.m. Detailed behavioral tests were described previously (22–25).

### Physiological assessments

#### Rotarod test

This test is used to assess motor coordination in mice (26). This study used an accelerating rotarod apparatus (Ugo Basile) for a maximum of 6 min. The tendency is for mice to stay on the rod to avoid falling, which is a measure of balance, grip strength and coordination. The rod was accelerated (the acceleration rate 6 rpm/min) from 4 to 40 revolutions per minute (RPM) in 6 min trial; three trials were conducted for each mouse on two different days. The latency to fall from the rod was recorded.

#### Catwalk

CatWalk^™^ XT (Noldus Information Technology, The Netherlands) is a gait analysis system for quantitative assessment of footprints and locomotion in rodents. After habituation for 30 min in the test room, the mouse walks across a tunnel (60 × 6 × 15 cm) from left to right (or right to left). CatWalk^™^ XT 9.1 software was used for the data analysis. Six runs were acquired in a trial for each mouse within 6 s per run; a run was regarded as compliant by the software if the maximum speed variation was less than 60%. Footprints on the glass plate were detected by the camera below and transferred to the computer. The most frequently used parameters in Catwalk: Swing Duration, Print Size, Stride Length, Max Contact Area (27), Regularity index (of step sequence), Base of support (28) and Average speed were analyzed.

#### Grip force

The test is used to measure the neuromuscular function as maximal muscle strength of forelimbs. The measuring instrument (ALMEMO^®^ 2490) has a T-bar connected to a sensor. Prior to the test, body weight of the mouse was measured for later data normalization. In the first step, the tail of the mouse is gently pulled, keeping the torso horizontal, and the mouse is trained to correctly grasp the T bar of the sensor. Once it is established that the mouse is well-trained, the maximum muscle strength is measured. Ten trials were performed with intervals for rest every five trials and the average of the three maximum values were normalized against mouse body weight (29).

#### Wire hang test

This test is a measure of neuromuscular function and motor coordination, which is based on the latency of a mouse to fall off a metal grid upon exhaustion. The mouse is placed on a large metal cage grid. After the mouse grasps the grid with four paws, the grid is inverted with the mouse’s head declining first, suspended 40 cm above the open field box (30). The latency to fall was recorded and the maximum cut-off time was 3 min.

#### Hot plate test

Nociception and heat thresholds in rodents are assessed with the hot plate test (31). The mouse is placed on a hot plate apparatus (19 diameter metal plate covered around by 25 cm height transparent cylinder, Ugo Basile), and the surface is maintained at a constant temperature of 52 ± 0.5°C. The latency of response is measured from the time the mouse is placed on the plate (*t* = 0) to the time when a nocifensive behavior (hind paw licking or withdrawal) occurs (32). Once the nocifensive behavior is observed, the mouse is immediately removed from the hot plate to prevent tissue damage. The maximum cut-off time was set at 30 s. Higher latency indicates lower nociception.

### Emotional behavior assessments

All emotional behavior assessments described below were monitored and recorded by a video camera, placed above the experiment chamber (or apparatus) to track the motion of the mouse. The camera was connected to the SMART Video Tracking software from Pan Lab, Harvard Apparatus (SMART system).

#### Open field test (OFT)

The OFT was used to assess locomotor activity and anxiety-like behavior. The mouse is placed in a novel chamber (40 × 40 × 40 cm) divided into two zones: center and peripheral (center is 36% area of the zone). The OFT was conducted as previously described (33). In short, the mouse is gently placed in the corner of the open field box under 150–200 lux light, and allowed to move freely for 10 min. The SMART system records and evaluates movement, total distance traveled, time spent in a zone, and number of entries to center. Remaining for a longer period of time in the peripheral of the open field is measured as ‘thigmotaxis (the tendency remaining close to the wall)’, which indicates an increased level of anxiety (33).

#### Elevated plus maze test

The elevated plus maze test is widely used for assessing anxiety-like behaviors in mice. The mouse is placed in the center (5 × 5 cm) of an elevated maze (43 cm height) with a ‘plus’ configuration, comprised of two open arms (30 × 5 cm) and two closed arms (30 × 5 × 13.5 cm, enclosed on three sides). The mouse is placed with its head directed towards a closed arm and allowed to explore freely for 5 min. The illumination level of the test room is maintained at 100 lux (34), with the open arms at 80–90 lux, closed arms at 0–20 lux. The video camera and SMART system recorded time spent in an open arm, and number of entries into arms. Time spent in zone and entries to zone was counted when the center of mass was inside the zone. Less time in the open arms or longer time in the closed arms indicates greater anxiety-like behavior.

#### Light/Dark box test

The light/dark box test is widely used as a measure of anxiety-like behavior, which is based on the natural tendency of mice to avoid bright light (35). A rectangular box is divided into two chambers (40 × 20 × 40 cm each) separated by a door: one with bright light (1000–2000 lux), and the other dark (2–5 lux). The mouse was placed in the dark chamber at first, and was allowed to explore freely for 5 min in both chambers. The SMART system recorded time spent in the chamber, the distance traveled in chamber, the entries to the light chamber, and total number of transitions. Less time in the light chamber indicates a higher level of anxiety.

#### Emergence test

The emergence test, adapted from the open field test, is designed to assess neophobia and exploratory behavior in mice. The test procedure was modified from a previous study (36). By providing a safe place (a small opaque box 12 × 7.5 × 8 cm with an exit 7.5 × 8 cm on one side) as a shelter within the center of the open field (40 × 40 × 40 cm), illuminated 30–40 lux, we evaluated novelty-based anxiety in a bright open space. The test begins by placing the mouse inside the box and then enclosing it with a lid. After 30 s, the lid is removed and the latency of the mouse to emerge from the box is recorded. Data also included total time spent in the box, number of entries to the box, and total distance in a 10-min session, which was measured by an observer with a stop-watch as well as the recordings from the SMART system.

#### Novelty suppressed feeding test

The use of hyponeophagia, in which exposure to a novel environment suppresses feeding behavior after food deprivation, is another means of assessing anxiety-like behavior (37). After 24 h of food deprivation, mice are transferred to a testing chamber for 1 h of habituation. The open field box (40 × 40 × 40 cm) is filled with 1–1.5 cm of the same bedding as in the home cage; a small food pellet (3–4 g) is placed on a white paper (12 cm diameter) in the center arena, which is illuminated with 2000–2500 of light, and 500–1000 lux in the periphery. Following habituation, the mouse is placed at the corner of arena, and allowed to move freely for 10 min (38). Times of sniffing and touching the food before eating and the latency of the first bite are recorded. Next, the mouse is placed alone in its home cage for 5 min with a 3–4 g food pellet placed in the food tray. Food consumption during the first 5 min and the latency of first bite is recorded. Longer latencies to approach and eat indicate higher levels of anxiety-like or depressive-like behavior.

#### Forced swim test

The forced swim test measures depressive-like behaviors in mice. The mouse is placed in a transparent glass cylinder (20 cm diameters × 50 cm height) filled with water (25 cm depth, temperature 23–25°C). Activity for each mouse is recorded with a video camera and tracked by the SMART system for 6 min in the environment with lighting of approximately 100 lux. The latency to immobility was recorded from the beginning to the first bout of immobility, while the last 4 min of video was used to analyze mouse activity. Mouse activity was classified into three types by different acceleration thresholds (300 cm/s^2^, 450 cm/s^2^) with the SMART system: immobility, low activity (swimming), and high activity (struggling) (39). Criteria for immobility is the mouse is floating for at least 1 s without any movements, except those necessary to maintain body balance and nose above water (40). Watching the videos with the SMART system tracking simultaneously, mouse acceleration of less than 300 cm/s^2^ was considered as meeting the criteria for immobility. Shorter latency to immobility and longer time immobile indicates higher levels of depressive-like behavior.

#### Fear conditioning test

Fear conditioning is an associative learning paradigm for measuring aversive learning and memory. We used a tone (2000 Hz, 80 dB) as the conditioned stimulus (CS) paired with a mild foot shock (0.4 mA, 2 s) as the unconditioned aversive stimulus (US) in the background of 60 dB white noise. The conditioning box (25 × 25 × 25 cm) contained with an electrifiable grid floor, which was in a soundproof chamber connected to a tone source (Fear interface LE 118-8, Panlab, Harvard Apparatus), a shock generator (Shocker LE 100-26, Panlab, Harvard Apparatus), and a load cell coupler (LE 111, Panlab, Harvard Apparatus). Two sessions were conducted in two days: Session 1 (Day 1) consisted of a Conditioning test (training) for 8 min; Session 2 (Day 2) is begun 24 h after completion of Session 1, which consists of a contextual test for 5 min. Session 1, the Conditioning test is comprised of a 2-min acclimation, followed by the CS (30 s tone; 2000 Hz, 80 dB) and the US (mild foot shock of 2 s; 0.4 mA); both stimuli are co-terminated within the last 2 s of the tone. After a 90 s interval, the same pairing of the CS and US is repeated three times in total (120, 240 and 360 s after the start of the test, 90 s intervals between the pairing and pre-end). Light is on and freezing behavior is monitored. The mouse is considered freezing when there is a complete lack of movement for at least 2 consecutive seconds (41). The Contextual test (Session 2) is conducted in the same box used for conditioning; the mouse is placed in the box and allowed to freely explore for 5 min with no stimuli. Light and background white noise is identical to Session 1. All tests were recorded with a video camera and PACKWIN (Panlab, Harvard Apparatus) software on a computer connected to the device, data were analyzed using the software in FREEZING mode.

### Social interactions, social dominance, learning and memory

A previous study demonstrated low illumination levels (<20 lux) may increase social investigation in mice (42). Therefore, to avoid the induction of stress by a bright environment, which might affect test results, all the following tests were conducted with illumination levels <20 lux.

#### Three-chambered social test

This test measures preferences for sociability and social novelty. The apparatus has three connected rectangular chambers (40 × 20 × 40 cm each): left, right, and center illuminated at 4–6 lux. For consistency with previous study (43), a 30-min training session was conducted, which exposed the mouse to the wire cup that would contain the novel mouse during the test session. During the first session, a mouse was placed in the center chamber of the empty apparatus and allowed to explore the three chambers freely for 10 min as a habituation period. After the first session, the mouse was allowed to test in the center chamber with both chamber doors closed and a novel mouse was placed inside one of the wire cups (8 cm diameter × 10 cm height) in the left or right chamber. A similar, but empty, wire cup was placed in the other chamber. The chamber where novel mouse was placed (left or right) was randomly arranged between different subject mice to avoid side preference. Session 2 was the sociability trial, which started by opening both chamber doors and letting the subject mouse explore the three chambers freely for 10 min. Sociability is determined by the amount of time the mouse spends exploring the empty chamber and the chamber with the novel mouse over 10 min. Session 3 places a new novel mouse into the chamber that was empty in Session 2, which makes the novel mouse from Session 2 the familiar mouse, and the social novelty test begins again. Sociability is analyzed by calculating a Social Preference Index using the formula: (Tn − Te)/(Tn + Te) (Tn: Time in novel mouse interaction zone; Te: Time in empty cup interaction zone, the interaction zone was about 3.5 cm around the cup). The Social Novelty Preference Index is calculated by the formula: (Tn − Tf)/(Tn + Tf) (Tn: Time in novel mouse interaction zone; Tf: Time in familiar mouse interaction zone). Activity was monitored by video recording and tracked by the SMART system during the three sessions. Longer time spent with the novel mouse indicates greater sociability and social novelty. Conversely, lower social preference index reveals social interaction deficits (44). In this test, the male and female mice were about 23–25 weeks old, and the novel mice were adult 14-week-old (male), and 8-week-old (female).

#### Tube dominance test

The tube dominance test measures aggression to identify deficits in social interactions in strains of transgenic mice. WT mice and *Rtl1^m+/p-^* mice were assessed by placing subjects at the opposite ends of a clear, narrow acrylic tube (66 cm length, inside diameter 2.6 cm, outside diameter 3 cm); the more dominant mouse will show a greater level of aggressive behavior to force the other mouse out of the tube. When one mouse has all four paws out of the tube, it is considered the loser; the mouse remaining in the tube is the winner and the more dominant mouse. Mice are habituated to the tube prior to the test by training them to walk through the tube for 10–15 min each day for 2 days. Mice were tested in the order of pairwise match for three trials per match. The number of wins is reported as a percentage of the total number of matches; a higher percentage suggests more social dominance and social motivation (45).

#### Novel object recognition test-2h

The Novel object recognition test (NORT) assesses learning and memory of mice based on the innate preference to explore a novel object rather than a familiar one (46). The NORT-2h uses 2-h intervals between Sessions 2 and 3. We conducted three sections were in 2 days. Session 1 (Habituation): the mouse was allowed to freely explore in an open field box (40 × 40 × 40 cm) for 5 min. Session 2 (Familiarization): the test began 24 h after Session 1; the mouse was placed at the corner of the box and allowed to freely explore for 10 min. The mouse was trained to become familiar with two identical objects (light bulbs, 4.5 cm diameter × 8 cm height) placed in opposite quadrants of the box center (13 cm from the wall). Session 3 (Test): 2 h later, one of the identical objects from the training phase was replaced with a novel object (plastic toy, 3–5 cm diameter × 8 cm height), and the mouse was allowed to freely explore between the familiar and novel object for 10 min. The box was illuminated at approximately 10–20 lux to minimize stress from bright lighting. The test was monitored by video camera and tracked by the SMART system. A discrimination index (47) was determined by calculating (Time with familiar object − Time with the novel object)/(Time with familiar object + Time with novel objects), the interaction zone is 4 cm around the object. A positive discrimination index value in test (Session 3) indicates more time with the novel object and better memory. Data analysis excluded the climbing time on the objects, and by using PVC tape around the upper side of the objects to prevent the mouse from climbing. Data was excluded if the discrimination index in Session 1 was >0.25 (preference for one of the same objects).

#### NORT-24h

The protocol for NORT-24h is identical to that described for NORT-2h, except for the time between Session 2 and Session 3, which was conducted 24 h after Session 2 for 10 min (Day 3). Calculation of the discrimination index was also the same way as for NORT-2h. In this study, short-term memory was tested 2 h after the training phase and long-term memory was tested 24 h after the training phase.

#### T-maze spontaneous alternation test

T-maze spontaneous alteration assesses exploratory behavior as a measure of cognition and repetitive/stereotype behaviors in rodents. Spontaneous alternation behavior is the willingness of the mouse to explore a novel environment, defined as a new arm of the maze rather than a familiar arm, which acquires spatial working memory. The protocol and details of the test were based on a previous study (48). The T-maze apparatus is composed of three arms, the start arm (47 × 12 × 16.5 cm) for entry, two goal arms form the T of the maze (left and right) (106 × 12 × 16.5 cm), and three doors. There are 10 trials for each mouse, five consecutive trials separated by 2 days and two choice runs for arms in each trial. The trial is initiated by placing the mouse in the entry arm with the door closed (start chamber). Next, the door is opened and the mouse is allowed to choose freely between the two goal arms (first choice). Once the mouse choses to enter an arm (whole body and tail inside the arm), the door is closed immediately, which requires the mouse to remain inside for 30 s. After 30 s, the mouse is gently removed from the goal arm and placed in the start chamber. The door is pended and the mouse is once again allowed to choose between the two goal arms (second choice). If the mouse chooses the other arm as the second choice, it receives a score = 1, a second choice of the same arm receives as score = 0. The percentage of alternation (number of turns into each goal) is calculated for 10 trials.

#### T-maze rewarded alternation test

The same apparatus was used as for the spontaneous alternation test. Alternation behavior can be reinforced by making the mouse hungry and rewarding the mouse a preferred food (48). Different from the spontaneous tests requiring the T maze to be novel to mice, the mouse needs to be habituated in the T maze prior to the test. One week before testing, mice are offered the preferred food (peanut butter) for tasting in their home cage at least twice, to eliminate hyponeophagia. The day before the test, all mice from a home cage are group habituated by placing them into the T maze with a sample of peanut butter in food wells, placed at the end of each goal arm with all doors open. Mice are allowed to freely explore for 3 min; this is repeated twice with 10 min intervals, cage by cage. Next, individual mouse habituation was done with the same steps as group habituation, one by one. Twenty-four hours before the test, food is restricted by providing mice with 1–1.2 g pellets per mouse. A total of 10 trials were conducted for each mouse in one day. The difference from the spontaneous test is that the trials were conducted in turns between mice to reduce the different latency of time for food restriction. All mice finished the first trial and then second trial started. Each trial consisted of two choice runs: a forced-choice run, in which the mouse was forced to enter an assigned arm (the other goal arm door closed) and consumed the reward (a bit of peanut buffer); the second free-choice run, in which the mouse was allowed to choose freely from both goal arms. First, the mouse was placed in the start chamber with food rewards placed at both ends of the goal arms. After the mouse entered the assigned arm, the arm door was closed immediately. Once the reward was consumed, the time spent in finishing the food was recorded and the mouse was removed from the arm to the start chamber. In the second run, the mouse chose freely between two goal arms without any doors closed. If the mouse remembers the entry arm in the first run, it will go to the opposite arm and the score = 1. However, the score = 0 if the mouse chooses the same arm. The rewarded alternation percentage was calculated by the scores from these 10 trials. The assigned arm in 10 trials was randomly arranged (left or right, not the same arm consecutively in two trials). If the time spent on exploring the finishing the reward exceeded 2 min the trial was evaluated as a failure and excluded from the analysis. Higher score and higher percentages of alternation indicate a good working memory; lower scores indicate a deficit.

### Electrophysiological measures

#### LC slice preparation

Brain slice preparation was modified based on previous procedures for hippocampal slice culture and patch-clamp electrophysiology (23–25), and adapted to incorporate the *N*-methyl-d-glucamine (NMDG) protective recovery method (49). Briefly, mice were deeply anesthetized with 5% isoflurane and immediately sacrificed. The brains were removed and instantly prepared for vibratome sections (Leica VT1200 S, Leica). Thereafter, two to three 250 μm coronal sections were cut and transferred to an ice-cold modified NMDG-HEPES dissection buffer containing (in mm): 92 NMDG, 2.5 KCl, 20 HEPES, 1.2 NaH_2_PO_4_, 30 NaHCO_3_, 10 MgSO_4_, 0.5 CaCl_2_, 3 Na-pyruvate, 2 thiourea, 5 Na-ascorbate and 26 D-glucose (pH 7.3; 300 mOsmol/kg). Slices were transferred to standard artificial cerebrospinal fluid (ACSF), oxygenated with 95% O_2_–5% CO_2_ and containing (in mm): 125 NaCl, 2.5 KCl, 2 CaCl_2_, 2 MgSO_4_, 1.25 NaH_2_PO_4_, 26 NaHCO_3_ and 26 D-glucose (pH 7.3; 295 ~ 300 mOsmol/kg). After 30 min incubation at 32°C, the incubation chamber was maintained at room temperature (22–25°C) until recording.

#### Patch clamping recording

Individual slices were transferred to an immersion-type recording chamber mounted on an upright microscope (Axio Examiner D1, Zeiss) and continuously perfused with oxygenated ACSF at a rate of 2–3 ml/min and maintained at 32°C. The LC nuclei were visualized under 10× magnification on an upright microscope and identified as a cluster-shaped area situated lateral to the fourth ventricle. Neurons within the LC were identified with a 40× differential interference contrast (DIC) water immersion lens using a CCD camera. Whole-cell patch-clamp recordings were conducted in current-clamp and voltage-clamp mode using borosilicate micropipettes with an internal filament (inner diameter: 0.86 mm, outer diameter: 1.5 mm, length: 10 cm; Sutter Instrument, USA). The micropipettes were pulled using a laser-based programmable microelectrode puller (P-2000, Sutter Instrument, USA) and filled with an internal solution containing (in mm): 115 K-gluconate, 20 KCl, 8 NaCl, 10 HEPES, 2 Mg-ATP, 0.3 Na_2_-GTP, 0.2 EGTA. Osmolality was set to 285–290 (mOsm) and pH to 7.3, and electrode tip resistance was 5–7 MΩ. Data were recoded using a MultiClamp 700B amplifier equipped with a Digidata 1440 A/D converter (Molecular Devices, USA). Signals were digitized at 10 kHz and filtered at 2 kHz Bessel low-pass filter. Current-clamp recordings were performed at −60 mV holding potential. Only cell access resistance lower than 20 MΩ were accepted. Data were analyzed offline by pClamp 10.7 acquisition software (Molecular Devices, USA).

### Statistical analysis

All data are presented as means ± standard error of the mean (SEM) with sample sizes (*n*) shown in figures or stated in the text. Statistical analyses were performed using SigmaPlot 11 (Systat Software). Normality tests (Shapiro–Wilk) and equal variance tests were run and passed (*P* > 0.05) before parametric statistical analyses were performed. Non-parametric statistical analyses were performed if normality and equal variance tests were not passed (*P* < 0.05).

## Supplementary Material

4_Supplementary_Material_ddac110

Table_S1_ddac110

Table_S2_ddac110

Table_S3_ddac110

Table_S4_ddac110
