# Supplementary material for: RTL1/PEG11 imprinted in human and mouse brain mediates anxiety-like and social behaviors and regulates neuronal excitability in the locus coeruleus
Source: Hum Mol Genet. 2022 May 14;31(18):3161–80. doi: 10.1093/hmg/ddac110 (PMC9476620; doi:10.1093/hmg/ddac110)
Supplement: 4_Supplementary_Material_ddac110 [file 4_supplementary_material_ddac110.zip › 4_Supplementary_Material_ddac110.pdf]

RTL1/PEG11 imprinted in human and mouse brain mediates anxiety-like and social behaviors and regulates neuronal excitability in the locus coeruleus

Ming-Yi Chou[1,†], Meng-Chuen Hu[1,†], Pin-Yu Chen[1,†], Chi-Lin Hsu[1,†], Ting-Yu Lin[1,†], Mao-Jia Tan[1,†], Chih-Yu Lee[1,2], Meng-Fai Kuo[3], Pei-Hsin Huang[4], Vin-Cent Wu[5], Shih-Hung Yang[3], Pi-Chuan Fan[6], Hsin-Yi Huang[4], Schahram Akbarian[7], Tsui-Han Loo[8], Colin L. Stewart[8], Hsiang-Po Huang[2], Susan Shur-Fen Gau[1,9], and Hsien-Sung Huang[1,*]

SUPPLEMENTARY MATERIAL

SUPPLEMENTARY TABLES

Table S1. Details of primer sequences. Please see attached Excel File.

Table S2. Details of human subjects. Please see attached Excel File.

Table S3. Details of *RTL1* SNPs. Please see attached Excel File.

Table S4. Schedules of mouse behavioral tests. Please see attached Excel File.

SUPPLEMENTARY FIGURES

Figure S1. Expression patterns of RTL1 protein in different organs and developmental stages of brain in female mice.

Figure S2. Verification of the indel mutation site of *Rtl1* in F1 generation of *Rtl1*$^{m+/p-}$ mice.

Figure S3. Body weight, brain weight, and ratio of brain to body weight from male and female *Rtl1*$^{m+/p+}$, *Rtl1*$^{m-/p+}$ *Rtl1*$^{m+/p-}$, and *Rtl1*$^{m-/p-}$ mice at postnatal day 28.

Figure S4. Body weight from neonatal to adult stages of male and female *Rtl1*$^{m+/p-}$ and *Rtl1*$^{m-/p+}$ mice.

Figure S5. Placenta weight from two different embryonic stages of *Rtl1*$^{m+/p-}$ and *Rtl1*$^{m-/p+}$ mice.

Figure S6. *Rtl1* is paternally, but not maternally, expressed in TH-expressing brain regions of mice.

Figure S7. *Rtl1* is not expressed in periglomerular cells of the olfactory bulb of mice.

Figure S8. *Rtl1* is paternally, but not maternally expressed in non-TH-expressing brain regions of mice.

Figure S9. Most RTL1-expressing cells are neurons in non-TH-expressing brain regions of mice.

Figure S10. Most RTL1-expressing cells in mouse LSD are neurons.

Figure S11. Most RTL1-expressing cells in the AHiPM, APir, and PB of mice are neurons.

Figure S12. Summary of *RTL1* imprinting status in human brain and adrenal gland.

Figure S13. Normal learning and memory in paternal *Rtl1* knockout mice.

Figure S14. Normal repetitive pattern and heat sensation in paternal *Rtl1* knockout mice.

Figure S15. Normal spontaneous firing in LC neurons from paternal *Rtl1* knockout mice.

Figure S16. Parameters of action potential in LC neurons from paternal *Rtl1* knockout mice.

Figure S17. Female paternal *Rtl1* knockout mice displayed increased delay onset time of action potential and inward currents and decreased neuronal excitability of LC neurons.

Figure S18. Imprinting status of *RTL1* was altered in hiPSCs-derived cortical neurons.

Figure S19. Validation of SNP status of *RTL1* in human cohorts.

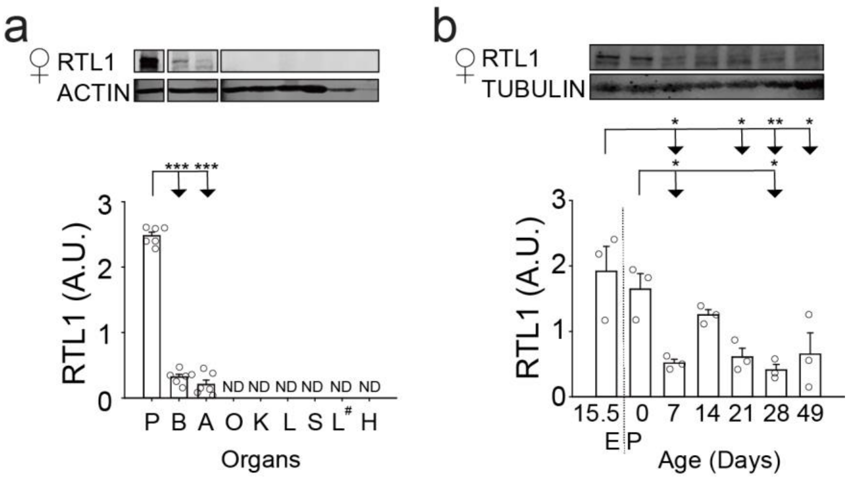

Figure S1. Expression patterns of RTL1 protein in different organs and developmental stages of brain in female mice. (**a**) Levels of RTL1 protein were measured with western blot analysis from different organs of P28 female mice. RTL1 protein levels were normalized to beta-ACTIN protein. One-way ANOVA with Holm-Sidak *post hoc* comparison. n = 6 samples. P = placenta, B = brain, A = adrenal gland, O = ovary, K = kidney, L = lung, S = spleen, $L^{\#}$ = liver, H = heart. (**b**) Levels of RTL1 protein were measured with western blot analysis from female mice at embryonic stage (embryonic day 15.5) to adult stage (postnatal day 49). RTL1 protein levels were normalized to alpha-TUBULIN protein. One-way ANOVA with Holm-Sidak *post hoc* comparison, *$P < 0.05$, **$P < 0.01$, ***$P < 0.001$, n = 3 brain hemispheres for each time points. All data are the mean ± s.e.m.

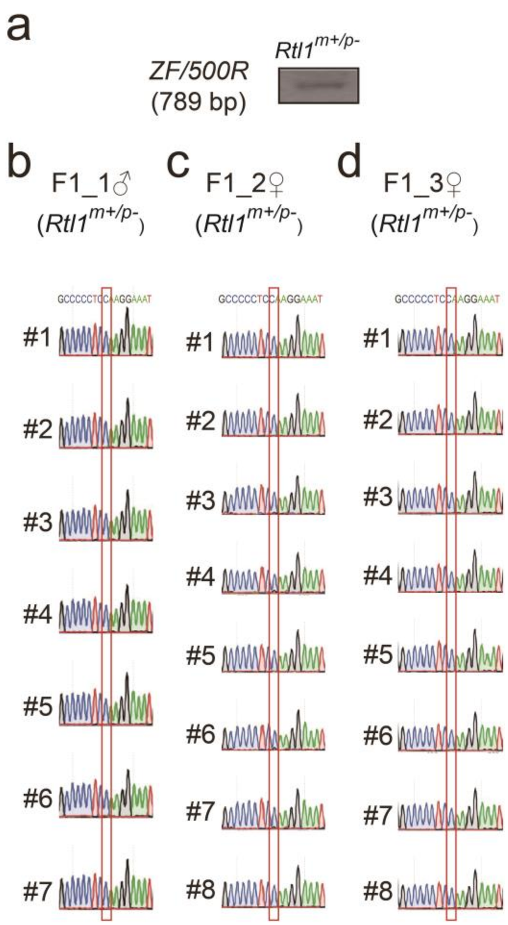

Figure S2. Verification of the indel mutation site of *Rtl1* in F1 generation of *Rtl1*$^{m+/p-}$ mice. (**a**) A 798 bp PCR product was generated from *Rtl1*$^{m+/p-}$ mice with ZF and 500R primers. (**b-d**) TA cloning was performed from three different *Rtl1*$^{m+/p-}$ mice of F1 generation. Sequences of each clone were verified with Sanger sequencing. n = 7 to 8 clones/mouse.

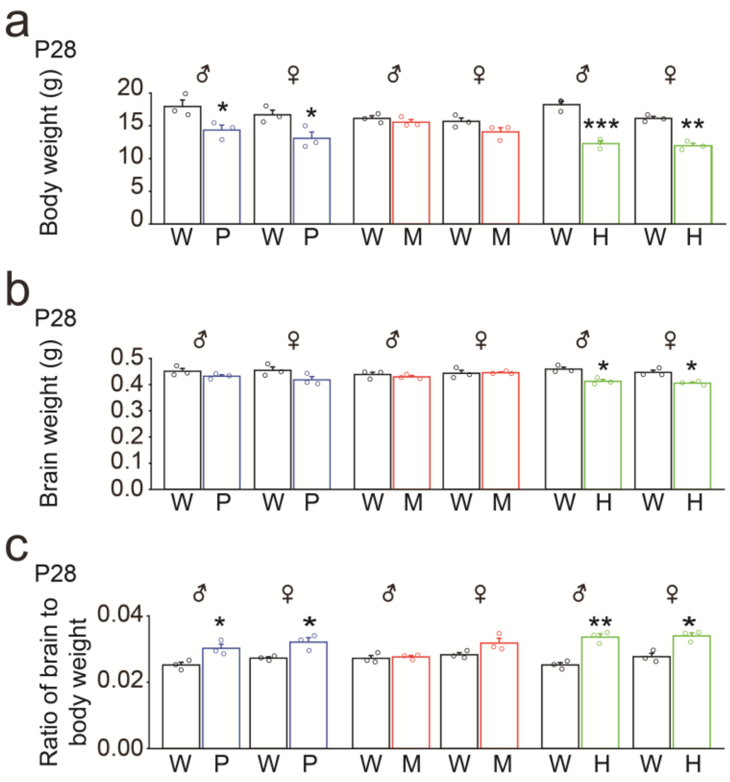

Figure S3. Body weight, brain weight, and ratios of brain to body weight from male and female *Rtl1*$^{m+/p+}$, *Rtl1*$^{m-/p+}$ *Rtl1*$^{m+/p-}$, and *Rtl1*$^{m-/p-}$ mice at postnatal day 28. Body weights (**a**), brain weights (**b**), and ratio of brain to body weight (**c**) were measured from male and female *Rtl1*$^{m+/p-}$ mice (P), *Rtl1*$^{m-/p+}$ mice (M), *Rtl1*$^{m-/p-}$ mice (H), and their corresponding WT controls (W) from postnatal day 28 (P28). Student's t-test, two-tailed, n = 3 per group. *$P < 0.05$, **$P < 0.01$, ***$P < 0.001$. All data are the mean ± s.e.m.

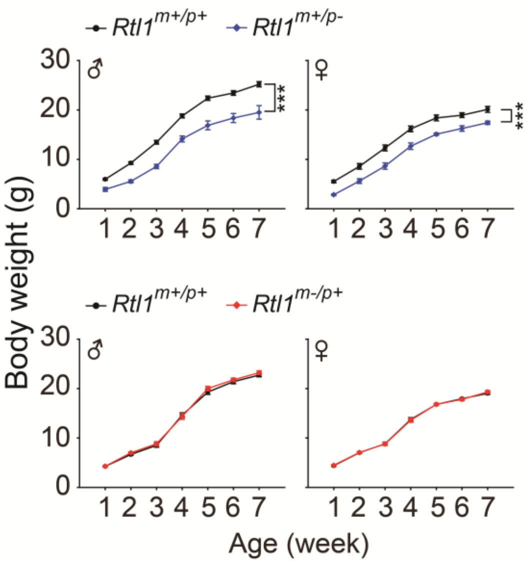

Figure S4. Body weight from neonatal to adult stages of male and female *Rtl1*$^{m+/p-}$ and *Rtl1*$^{m-/p+}$ mice. Body weights were measured from male and female *Rtl1*$^{m+/p-}$ mice (top), *Rtl1*$^{m-/p+}$ mice (bottom), and their corresponding WT controls from postnatal week 1 to 7. Two-way ANOVA with Holm-Sidak *post hoc* comparison, ***$P < 0.001$, n = 4 to 46 per group. All data are the mean ± s.e.m.

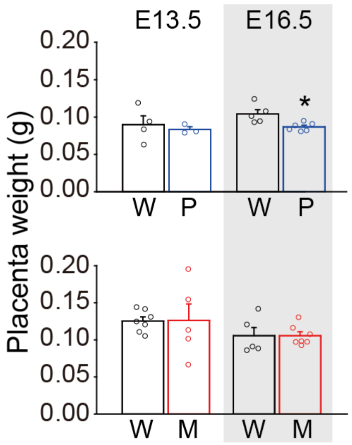

Figure S5. Placenta weight from two different embryonic stages of *Rtl1*$^{m+/p-}$ and *Rtl1*$^{m-/p+}$ mice. Weights of the placenta from *Rtl1*$^{m+/p-}$ mice (P) (top), *Rtl1*$^{m-/p+}$ mice (M) (bottom), and their corresponding WT controls (W) at E13.5 and E16.5. Student's t-test, two-tailed, *$P < 0.05$, n = 3 to 6 per group. All data are the mean ± s.e.m.

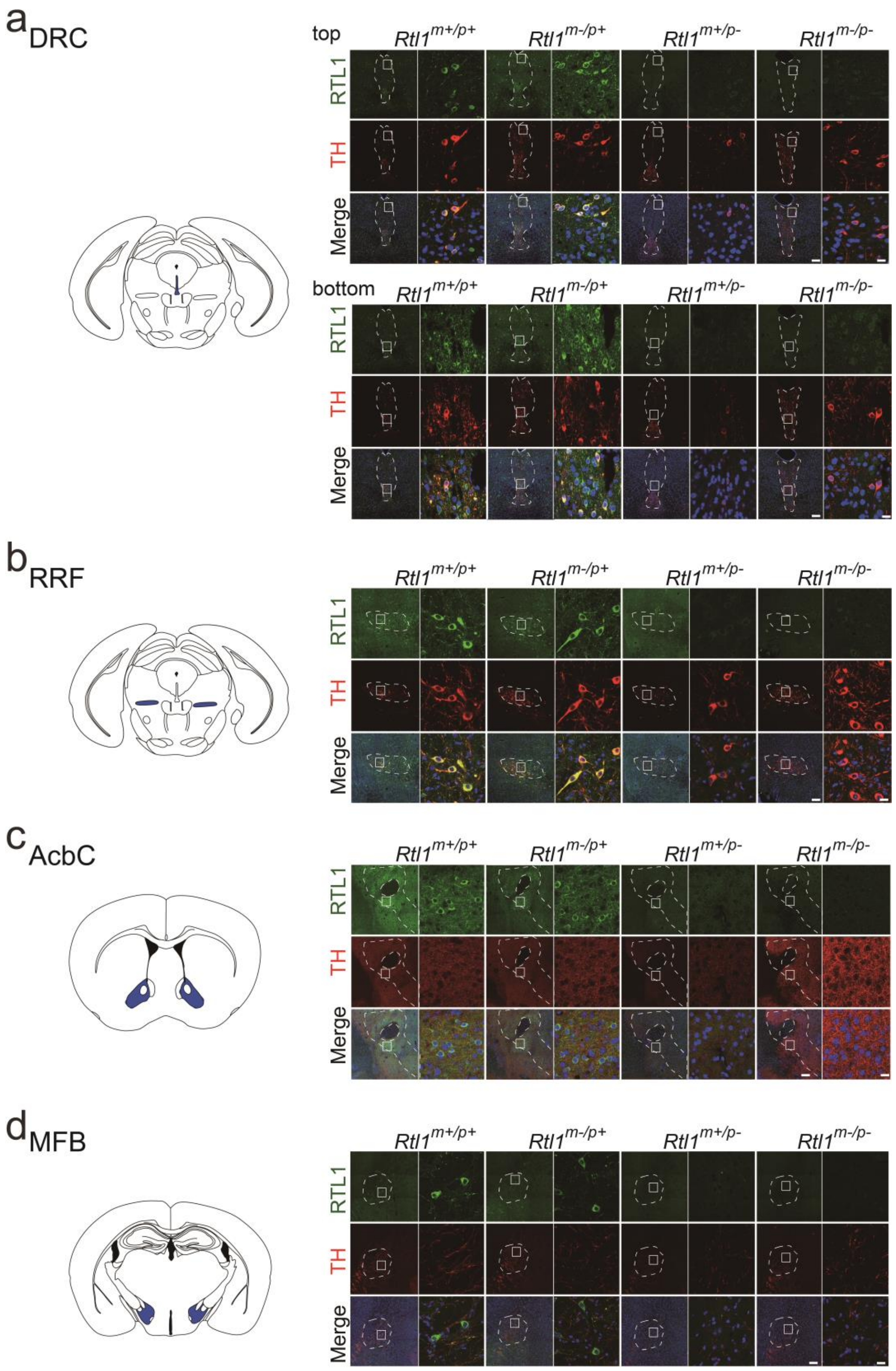
a DRC
top
bottom
$Rtl1^{m+/p+}$
$Rtl1^{m-/p+}$
$Rtl1^{m+/p-}$
$Rtl1^{m-/p-}$
RTL1
TH
Merge
b RRF
c AcbC
d MFB

Figure S6. *Rtl1* is paternally, but not maternally, expressed in TH-expressing brain regions of mice. Schematic diagram of TH-expressing brain regions containing paternally-expressed RTL1: (**a,** left) dorsal raphe nucleus (DRC), (**b,** left) retrorubral field (RRF), (**c,** left) accumbens nucleus core (AcbC), and (**d,** left) medial forebrain bundle (MFB). (**a-d,** right). Representative sections from mouse brains immunostained with RTL1 antibody (green), TH antibody (red), and counter-stained with DAPI (blue) in *Rtl1*$^{m+/p+}$, *Rtl1*$^{m-/p+}$, *Rtl1*$^{m+/p-}$, and *Rtl1*$^{m-/p-}$ mice. Scale bar = 150 µm. Squares indicate areas enlarged in left panel for each mouse genotype. Scale bar = 15 µm.

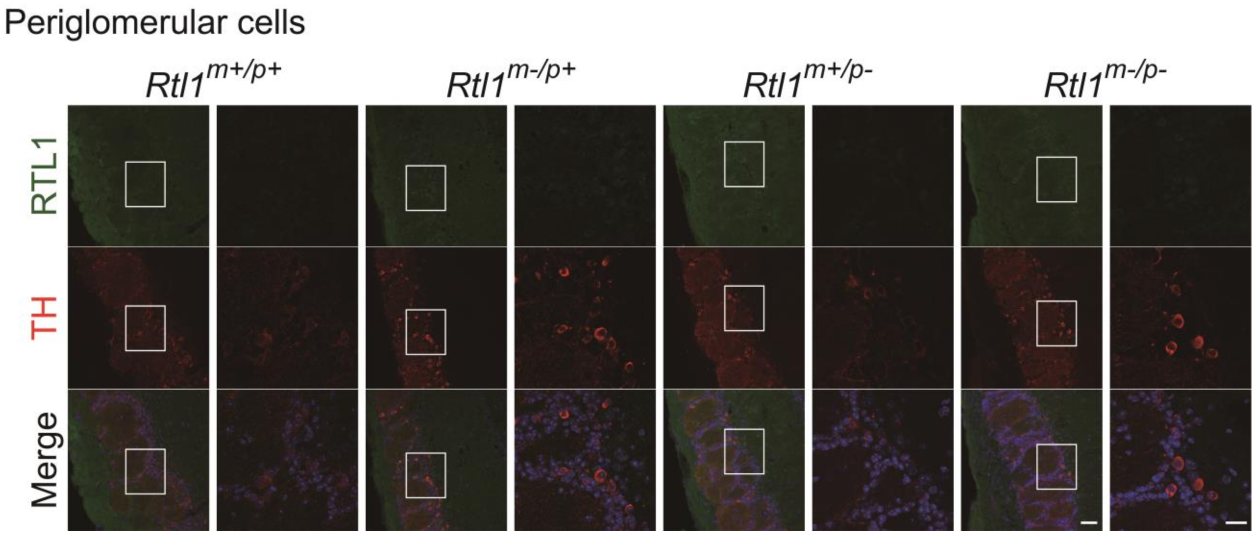

Figure S7. *Rtl1* is not expressed in periglomerular cells of the olfactory bulb of mice. Representative sections from mouse periglomerular cells immunostained with RTL1 antibody (green), TH antibody (red), and counter-stained with DAPI (blue) in *Rtl1*$^{m+/p+}$, *Rtl1*$^{m-/p+}$, *Rtl1*$^{m+/p-}$, and *Rtl1*$^{m-/p-}$ mice. Scale bar = 150 μm. Squares indicate areas enlarged in left panel for each mouse genotype. Scale bar = 20 μm.

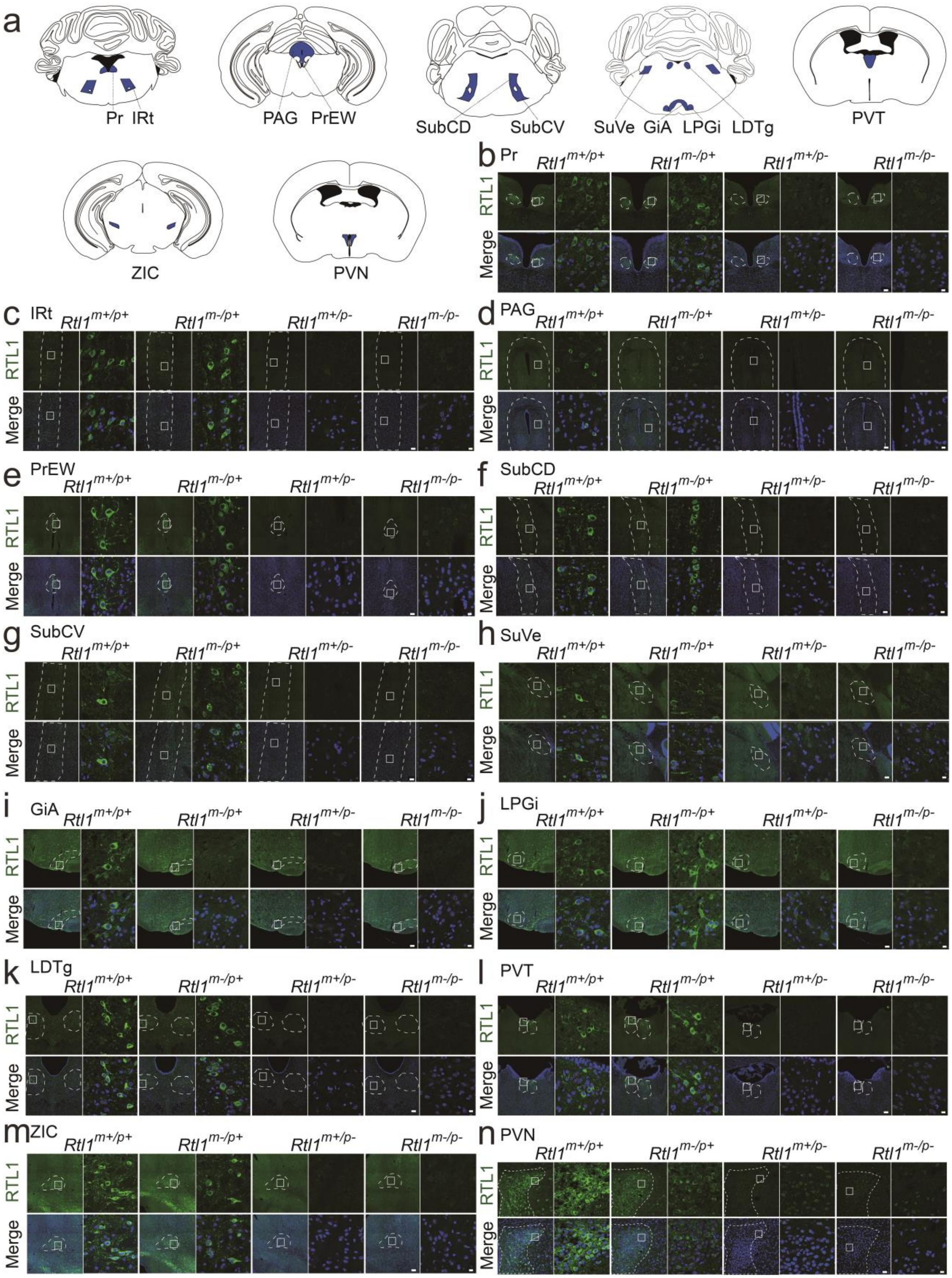
a
Pr
IRt
PAG
PrEW
SubCD
SubCV
SuVe
GiA
LPGi
LDTg
PVT
ZIC
PVN
b
c
d
e
f
g
h
i
j
k
l
m
n
Rtl1m+/p+
Rtl1m-/p+
Rtl1m+/p-
Rtl1m-/p-
RTL1
Merge

Figure S8. *Rtl1* is paternally, but not maternally, expressed in non-TH-expressing brain regions of mice. (**a**) Schematic diagram of non-TH-expressing brain regions containing paternally-expressed RTL1: prepositus nucleus (Pr), intermediate reticular nucleus (IRt), periaqueductal gray (PAG), pre-Edinger-Westphal nucleus (PrEW), subcoeruleus nucleus, dorsal part (SubCD), subcoeruleus nucleus, ventral part (SubCV), superior vestibular nucleus (SuVe), gigantocellular reticular nucleus, alpha part (GiA), lateral paragigantocellular nucleus (LPGi), laterodorsal tegmental nucleus (LDTg), paraventricular nucleus of the thalamus (PVT), zona incerta, caudal (ZIC), and periventricular nucleus (PVN). (**b-n**) Representative sections from mouse brains immunostained with RTL1 antibody (green), and counter-stained with DAPI (blue) in *Rtl1*$^{m+/p+}$, *Rtl1*$^{m-/p+}$, *Rtl1*$^{m+/p-}$, and *Rtl1*$^{m-/p-}$ mice: (**b**) Pr, (**c**) IRt, (**d**) PAG, (**e**) PrEW, (**f**) SubCD, (**g**) SubCV, (**h**) SuVe, (**i**) GiA, (**j**) LPGi, (**k**) LDTg, (**l**) PVT, (**m**) ZIC, and (**n**) PVN. Scale bar = 100 μm. Squares indicate areas enlarged in left panel for each mouse genotype. Scale bar = 20 μm.

1

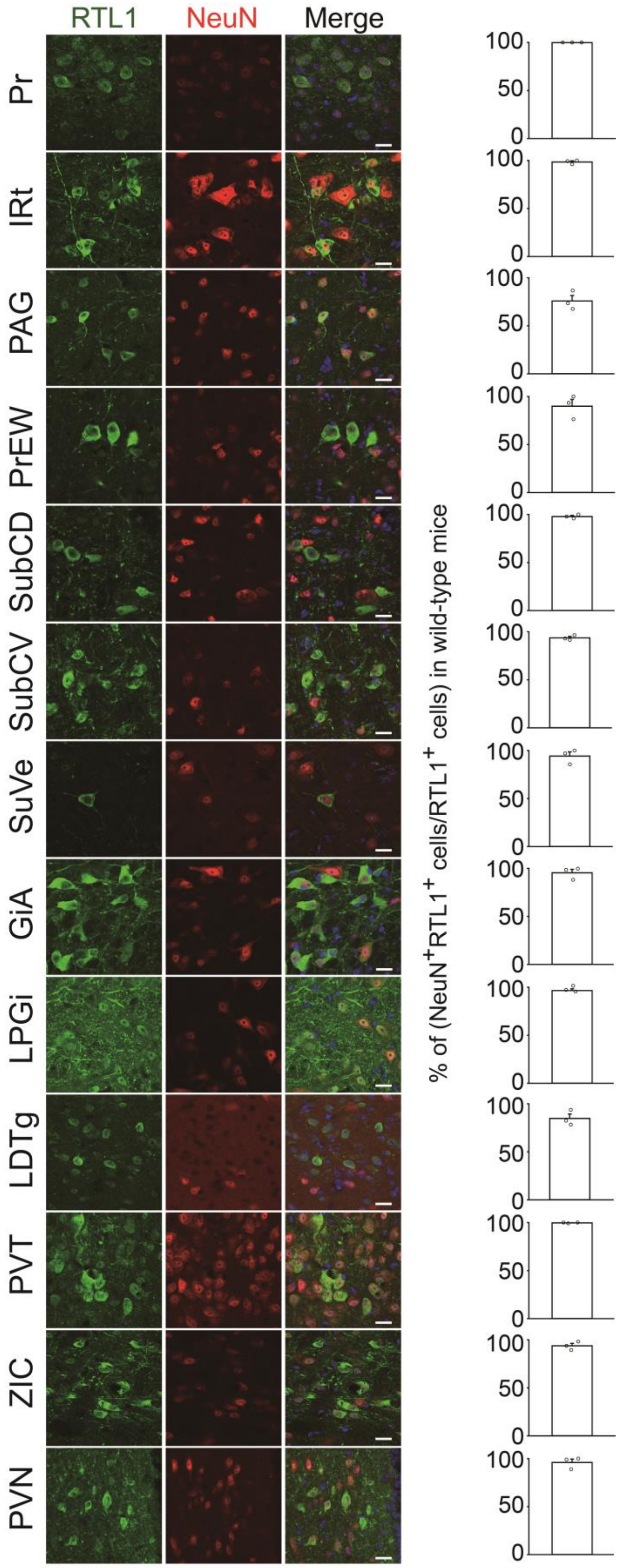

RTL1
NeuN
Merge
Pr
IRt
PAG
PrEW
SubCD
SubCV
SuVe
GiA
LPGi
LDTg
PVT
ZIC
PVN
% of (NeuN+RTL1+ cells/RTL1+ cells) in wild-type mice
100
50
0

Figure S9. Most RTL1-expressing cells are neurons in non-TH-expressing brain regions of mice. (Left) Representative sections from mouse brains immunostained with RTL1 antibody (green), NeuN antibody (red), and counter-stained with DAPI (blue). (Right) Bar graphs indicate the percentage of neuronal cell types in RTL1-expressing cells from non-TH-expressing brain regions of mice: prepositus nucleus (Pr), intermediate reticular nucleus (IRt), periaqueductal gray (PAG), pre-Edinger-Westphal nucleus (PrEW), subcoeruleus nucleus, dorsal part (SubCD), subcoeruleus nucleus, ventral part (SubCV), superior vestibular nucleus (SuVe), gigantocellular reticular nucleus, alpha part (GiA), lateral paragigantocellular nucleus (LPGi), laterodorsal tegmental nucleus (LDTg), paraventricular nucleus of the thalamus (PVT), zona incerta, caudal (ZIC), and periventricular nucleus (PVN). Scale bar = 20 μm. n = 3 per group. WT: wild-type mice. All data are the mean ± s.e.m.

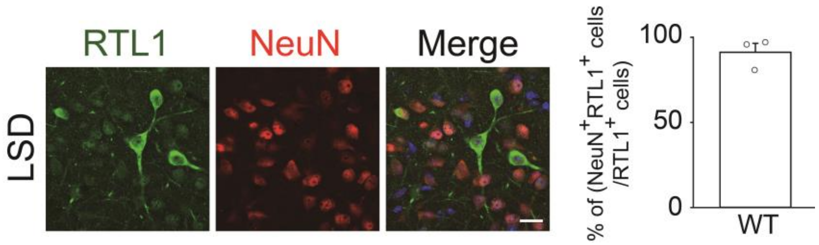

Figure S10. Most RTL1-expressing cells in mouse LSD are neurons. (Left) Representative sections from the lateral septal nucleus, dorsal part (LSD) of mouse immunostained with RTL1 antibody (green), NeuN antibody (red), and counter-stained with DAPI (blue). (Right) Bar graphs indicate the percentage of neuronal cell types in RTL1-expressing cells from mouse LSD. Scale bar = 20 μm. n = 3 per group. WT: wild-type mice. All data are the mean ± s.e.m.

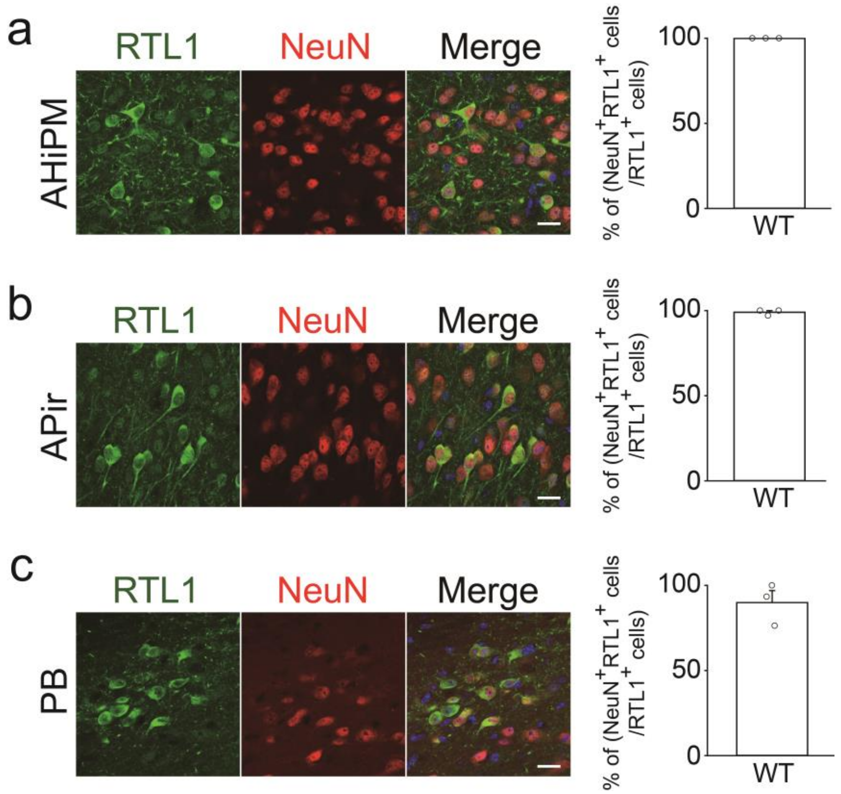

Figure S11. Most RTL1-expressing cells in the AHiPM, APir, and PB of mice are neurons. (Left) Representative sections from mouse brains immunostained with RTL1 antibody (green), NeuN antibody (red), and counter-stained with DAPI (blue). (Right) Bar graphs indicate the percentage of neuronal cell types in RTL1-expressing cells from the AHiPM, APir, and PB of mice: (**a**) amygdalohippocampal area, posteromedial part (AHiPM), (**b**) amygdalopiriform transition (APir), and (**c**) parabrachial nuclei (PB). Scale bar = 20 μm. n = 3 per group. WT: wild-type mice. All data are the mean ± s.e.m.

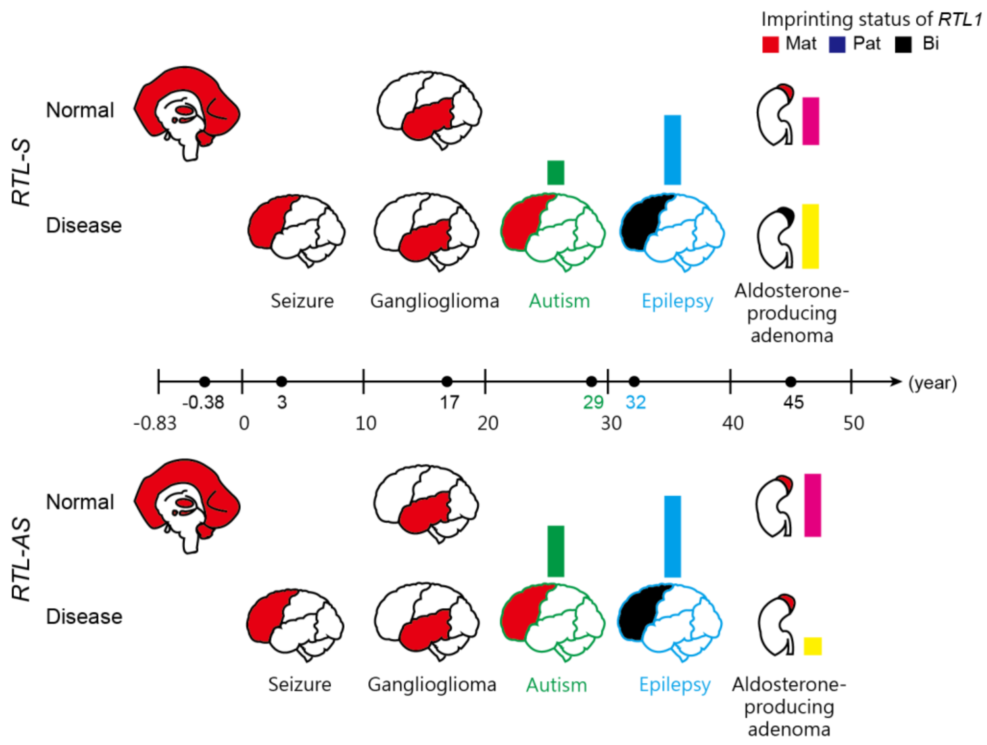

Figure S12. Summary of *RTL1* imprinting status in human brain and adrenal gland. Schematic diagram of *RTL1* imprinting status across different developmental stages and in different tissues. RNA expression levels of *RTL1* are also shown for postmortem or surgical resection tissues from 29-year-old, 32-year-old, and 45-year-old subjects.

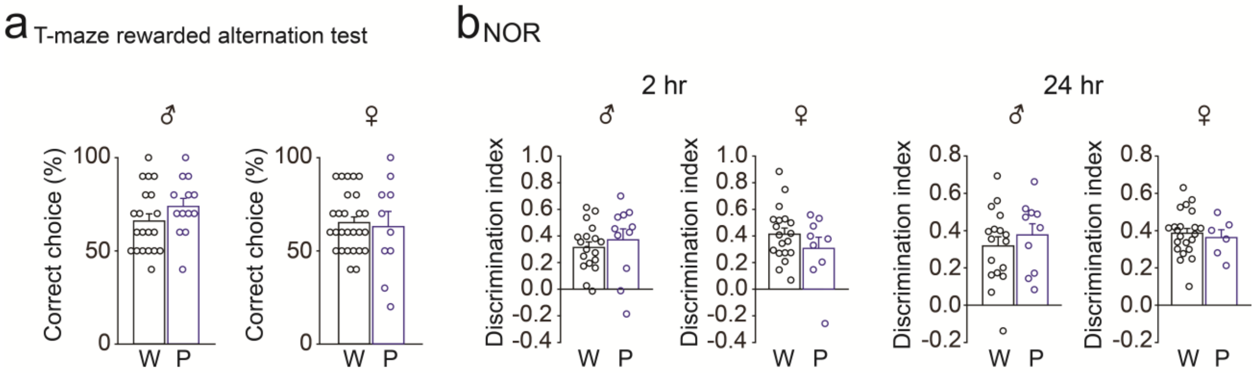

Figure S13. Normal learning and memory in paternal *Rtl1* knockout mice. (**a**) Bar graph indicting the percentage of correct choices. (**b**) Bar graph indicating the discrimination index after 2 hr (left) and 24 hr (right). Wild-type mice (W); paternal *Rtl1* knockout mice (P). W (male), n = 17-20; P (male), n = 10-13; W (female), n = 19-27; P (female), n = 6-10. All data are the mean ± s.e.m.

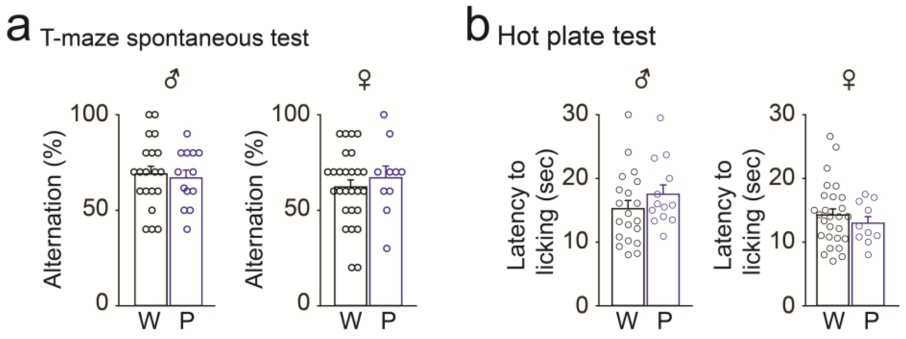

Figure S14. Normal repetitive pattern and heat sensation in paternal *Rtl1* knockout mice. (**a**) Bar graph showing percentage of alternation. (**b**) Bar graph indicating latency to licking paws. *Rtl1*$^{m+/p+}$ mice (W); *Rtl1*$^{m+/p-}$ mice (P). W (male), n = 20; P (male), n = 13; W (female), n = 27; P (female), n = 10. All data are the mean ± s.e.m.

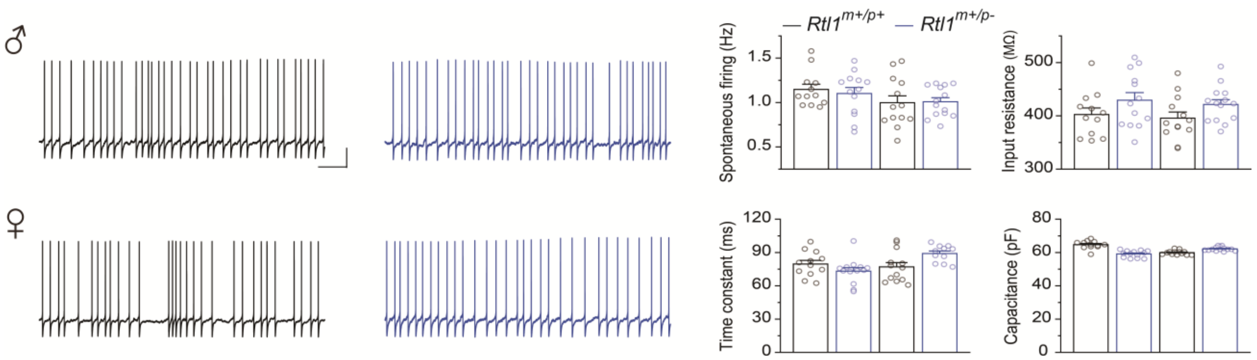

Figure S15. Normal spontaneous firing in LC neurons from paternal *Rtl1* knockout mice. (Left) Representative traces of spontaneous firing patterns were obtained in current-clamp recording from LC neurons in conditions of physiological temperature. Black traces represent *Rtl1*$^{m+/p+}$ mice; blue traces represent *Rtl1*$^{m+/p-}$ mice. Scale bar represents 20 mV/3 msec. (Right) Bar graphs showing spontaneous firing rates, input resistance, membrane time constant and capacitances from LC neurons of *Rtl1*$^{m+/p+}$ mice (n = 12 and 13 slices from 7 male and 7 female mice, respectively) and *Rtl1*$^{m+/p-}$ mice (n = 13 and 14 slices from 7 male and 7 female mice, respectively). All data are the mean ± s.e.m.

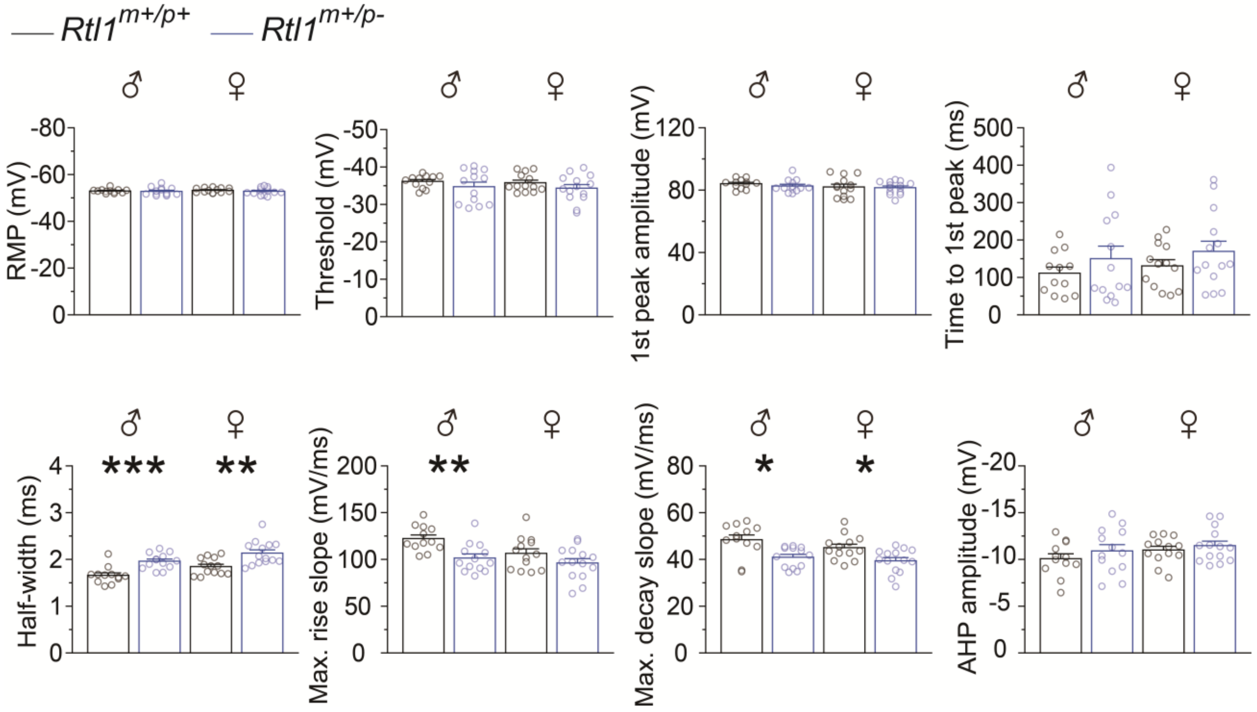

Figure S16. Parameters of action potential in LC neurons from paternal *Rtl1* knockout mice. Bar graphs show the resting membrane potential (RMP), threshold potentials, the first action potential amplitudes, time to peak of first action potentials, half-width, maximin rise slope for first action potentials, maximum decay slope of first action potentials, and the after-hyperpolarization potential (AHP) amplitudes in response to 30 pA current injection from LC neurons. Student's t-test, two-tailed, **P* < 0.05, ***P* < 0.01, ****P* < 0.001. *Rtl1*$^{m+/p+}$ mice (black bars), n = 12 and 13 slices from 7 male and 7 female mice, respectively; *Rtl1*$^{m+/p-}$ mice (blue bars), n = 13 and 14 slices from 7 male and 7 female mice, respectively). All data are the mean ± s.e.m.

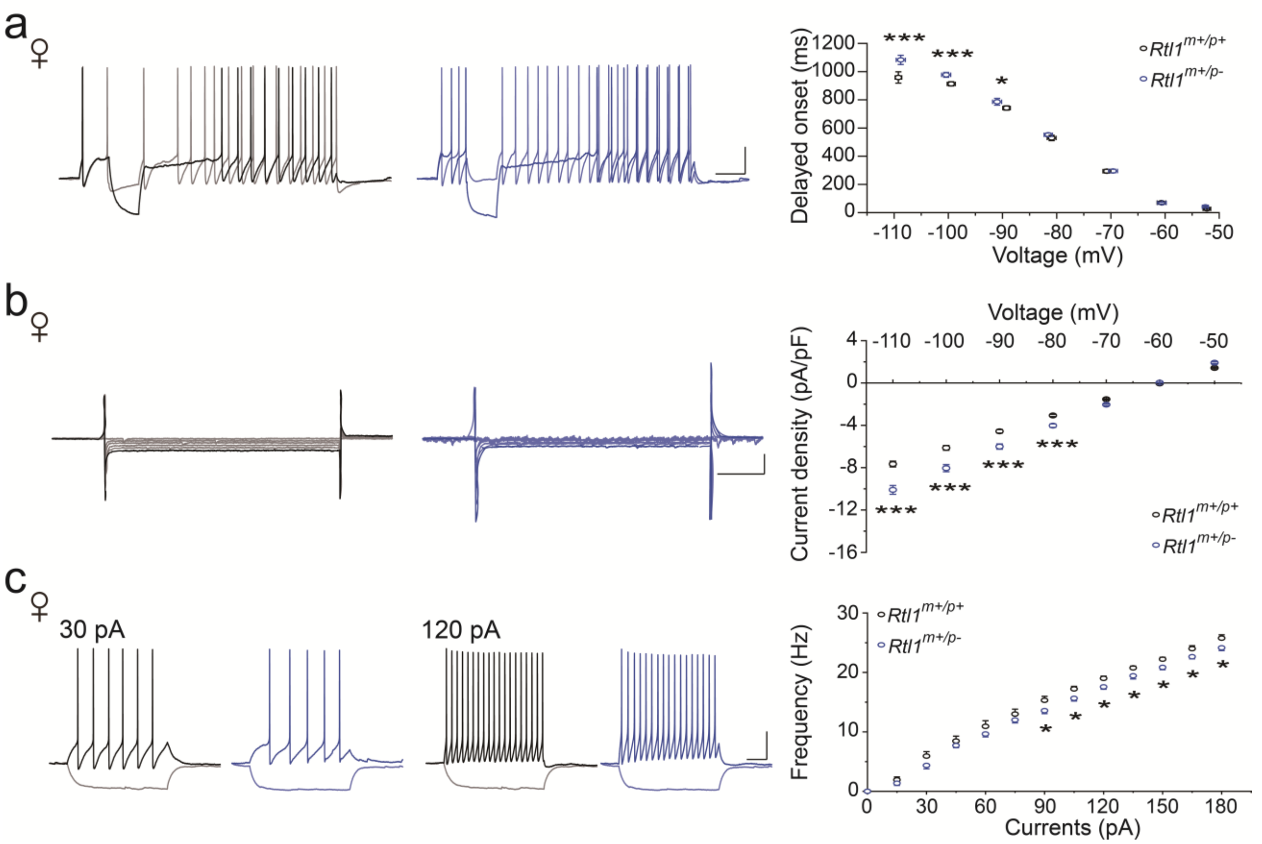

Figure S17. Female paternal *Rtl1* knockout mice displayed increased delay onset time of action potential and inward currents and decreased neuronal excitability of LC neurons. (a) Left: Representative current clamp recording traces from LC neurons of *Rtl1*$^{m+/p+}$ mice (black), and *Rtl1*$^{m+/p-}$ mice (blue) demonstrating hyperpolarization of the membrane voltage prolongs the "onset" of action potentials. The dark line represents depolarization at -60 mV and the light line represents depolarization at -110 mV. Scale bar represents 20 mV/300 msec. Right: Graph of the relationship between delay time and membrane voltage (V_m) shows the V_m-dependent manner of delayed excitation of LC neurons. (**b**) Left: Representative traces evoked from LC neurons in response to voltage steps from −50 to −110 mV. Scale bar represents 150 pA/100 msec. Right: Graph of current-voltage relationships showing the linear current responses to hyperpolarizing voltage from LC neurons. (**c**) Left: Representative traces from LC neurons of *Rtl1*$^{m+/p+}$ mice (black) and *Rtl1*$^{m+/p-}$ mice (blue) demonstrating responses to different current injections. Scale bar

represents 20 mV/200 msec. Right: Graph showing quantification of frequency of action potential firing with current injections of increasing magnitude. Two-way repeated measures ANOVA with Holm-Sidak *post hoc* comparison, $*P < 0.05$, $***P < 0.001$. *Rtl1*$^{m+/p+}$ mice, n = 13 slices from 7 female mice; *Rtl1*$^{m+/p-}$ mice, n = 14 slices from 7 female mice. All data are the mean ± s.e.m.

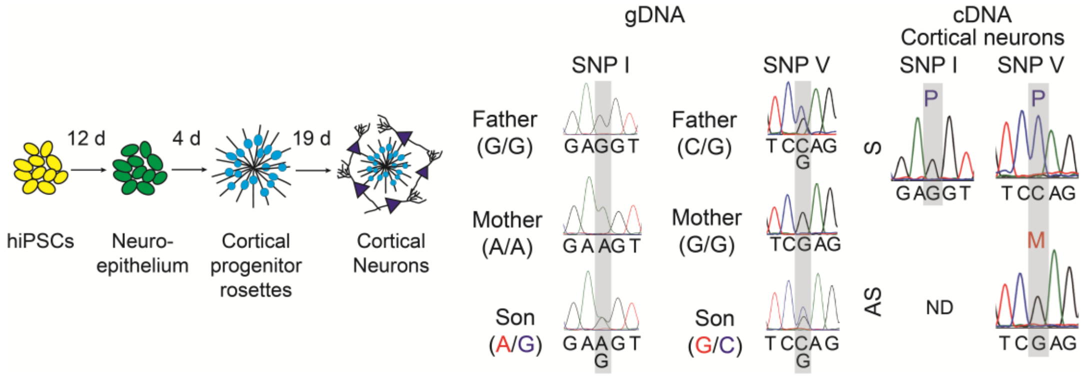

Figure S18. Imprinting status of *RTL1* was altered in hiPSCs-derived cortical neurons. Schematic diagram of the timeline for differentiation of hiPSC-derived cortical neurons examined in this study (left). SNP sites on the *RTL1* locus with known parental origin of each allele were identified by Sanger sequencing of genomic DNA extracted from blood samples of the family trio (middle). Parent-of-origin-specific allelic expression of *RTL1* in the hiPSC-derived cortical neurons was determined by Sanger sequencing (right). “P” = paternal expression. “M” = maternal expression. “ND” = non-detectable.

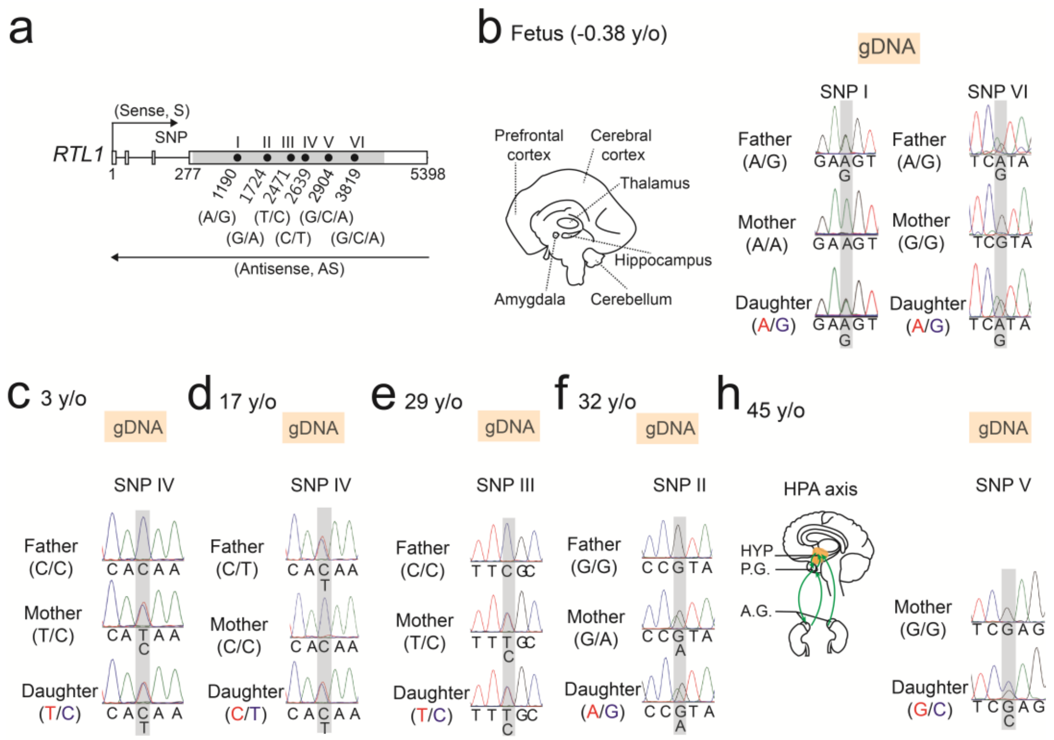

Figure S19. Validation of SNP status of *RTL1* in human cohorts. (**a**) Schematic diagram of SNP sites of *RTL1* used in human cohorts. SNP sites of *RTL1* were validated by Sanger sequencing in human cohorts (**b-h**).
